# Osteoclast-derived microRNA-containing exosomes selectively inhibit osteoblast activity

**DOI:** 10.1038/celldisc.2016.15

**Published:** 2016-05-31

**Authors:** Weijia Sun, Chenyang Zhao, Yuheng Li, Liang Wang, Guangjun Nie, Jiang Peng, Aiyuan Wang, Pengfei Zhang, Weiming Tian, Qi Li, Jinping Song, Cheng Wang, Xiaolong Xu, Yanhua Tian, Dingsheng Zhao, Zi Xu, Guohui Zhong, Bingxing Han, Shukuan Ling, Yan-Zhong Chang, Yingxian Li

**Affiliations:** 1 State Key Laboratory of Space Medicine Fundamentals and Application, China Astronaut Research and Training Center, Beijing, China; 2 Key Laboratory of Animal Physiology, Biochemistry and Molecular Biology of Hebei Province, Key Laboratory of Molecular and Cellular Biology of Ministry of Education, College of Life Science, Hebei Normal University, Shijiazhuang, China; 3 College of Life Sciences, University of Chinese Academy of Sciences, Beijing, China; 4 Institute of Orthopedics, 309 Hospital of Chinese People’s Liberation Army, Beijing, China; 5 Key Laboratory of Chinese Academy of Sciences for Biomedical Effects of Nanomaterials and Nanosafety, National Center for Nanoscience and Technology of China, Beijing, China; 6 Institute of Orthopedics, General Hospital of Chinese People’s Liberation Army, Beijing, China; 7 Bio-X Center, School of Life Science and Technology, Harbin Institute of Technology, Harbin, China

**Keywords:** exosome, miRNA, osteoblast, osteoclast

## Abstract

MicroRNAs have an important role in bone homeostasis. However, the detailed mechanism of microRNA-mediated intercellular communication between bone cells remains elusive. Here, we report that osteoclasts secrete microRNA-enriched exosomes, by which miR-214 is transferred into osteoblasts to inhibit their function. In a coculture system, inhibition of exosome formation and secretion prevented miR-214 transportation. Exosomes specifically recognized osteoblasts through the interaction between ephrinA2 and EphA2. In osteoclast-specific miR-214 transgenic mice, exosomes were secreted into the serum, and miR-214 and ephrinA2 levels were elevated. Therefore, these exosomes have an inhibitory role in osteoblast activity. miR-214 and ephrinA2 levels in serum exosomes from osteoporotic patients and mice were upregulated substantially. These exosomes may significantly inhibit osteoblast activity. Inhibition of exosome secretion via *Rab27a* small interfering RNA prevented ovariectomized-induced osteoblast dysfunction *in vivo*. Taken together, these findings suggest that exosome-mediated transfer of microRNA plays an important role in the regulation of osteoblast activity. Circulating miR-214 in exosomes not only represents a biomarker for bone loss but could selectively regulate osteoblast function.

## Introduction

Bones undergo constant turnover throughout our lifespan. Bone homeostasis is precisely controlled and orchestrated by bone-forming osteoblasts, bone-resorbing osteoclasts and osteocytes. Intercellular communication among these cell types is crucial for the establishment and maintenance of bone remodeling [1–5]. Osteoblast regulation of osteoclasts is mediated mainly by tight interplay among receptor activator for nuclear factor-κB ligand (RANKL) and osteoprotegerin, which are produced by osteoblasts, and receptor activator for nuclear factor-κB (RANK), which is expressed on osteoclasts [6, 7]. The EPHB4-EFNB2-mediated switch from bone resorption to bone formation [8] and inhibition of bone formation by Sema4D-Plexin-B1 via RhoA [9] are also important coupling factors involved in this process. Osteoclast-to-osteoblast crosstalk occurs mostly through growth factors, such as transforming growth factor-β. Some soluble factors, such as sphingosine-1-phosphate, ephrins and semaphorins, are also involved in the regulatory effects of osteoclasts on osteoblasts [10]. Bidirectional signaling between osteoclasts and osteoblasts mediated by Eph tyrosine kinase receptors and ligands is also an important regulator during different phases of bone remodeling [11].

In bones, microRNAs (miRNAs) regulate multiple processes, including osteoblast and osteoclast differentiation, orchestration of bone programming and management of cell fate [12]. miRNAs were first determined to be present in the cytoplasm. Recent studies have shown that they also exist in the extracellular space [13–15]. These extracellular miRNAs are protected from RNase degradation, mainly due to their encapsulation in lipid-bilayered exosomes. Exosomes, small vesicles secreted by a multitude of cell types [16–22], are important mediators of intercellular communication in immune signaling [23], tumor survival [24], stress responses [25] and angiogenesis [26, 27]. The ability of exosomes to incorporate and transfer miRNAs indicates their ability to influence the physiological behavior of recipient cells [28, 29]. They also have important roles in various biological processes [30–33], including cancer progression, immune regulation and tissue repair. However, their roles in bone remodeling have not yet been reported.

Exosomes act in a paracrine or endocrine manner to facilitate a diversity of intracellular or intercellular signaling mechanisms. Once exosomes are released, they can either target a neighboring cell or reach cells of distant organs after entering the blood stream [34, 35]. The transfer of material is a highly specific process depending on the type and status of the donor and recipient cells and has various functional consequences. However, the exact mechanisms by which target cells take up and integrate materials carried by exosomes are poorly understood.

In this study, we discovered a novel mode of osteoclast–osteoblast communication. Overall, we found that osteoclasts secreted miRNA-enriched exosomes, and osteoclast-derived exosomes preferentially transferred miR-214 into osteoblasts to inhibit their activity. EphrinA2, carried by these exosomes, mediated the recognition of exosome by osteoblasts. Moreover, osteoclast-specific miR-214 transgenic mice (OC-TG214) exhibited downregulated osteoblast activity and increased levels of circulating miR-214. Levels of miR-214 encapsulated in exosomes and those of ephrinA2 on the exosome membrane were highly upregulated in OC-TG214 mice, osteoporotic patients and ovariectomized (OVX) mice. Exosomes from these mice displayed a marked inhibitory role on osteoblast activity. Therefore, inhibition of exosome release may alleviate OVX-induced osteoblast activity. These results not only demonstrate the role of exosomes in communication between osteoblasts and osteoclasts but also reveal a potential biomarker for diagnostic purposes.

## Results

### Characterization of exosomes secreted during osteoclastogenesis

**Figure 1:**
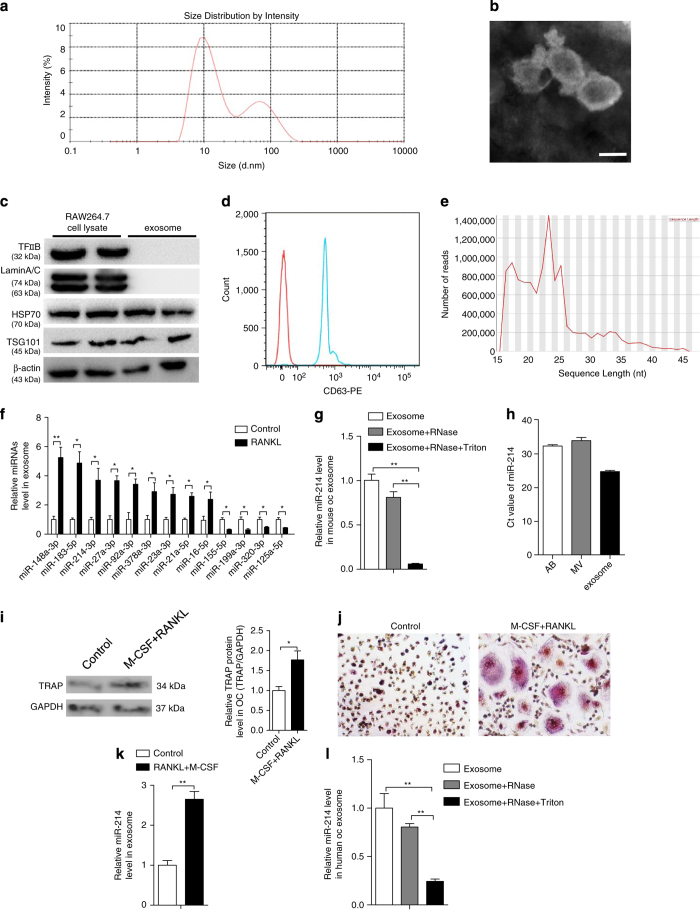
Identification and characterization of osteoclast-derived exosomes. (**a**) Size distribution of vesicles secreted by RANKL-induced RAW 264.7 cells for 2 days determined by dynamic light scattering. Data represent 20 measurements of four biological samples. (**b**) An electron microscopy image of exosomes. Scale bar, 100 nm. (**c**) The protein levels of TFIIB, LaminA/C, HSP70 and TSG101 in RAW 264.7 cell lysates and exosomes secreted by RANKL-induced RAW 264.7 cells were analyzed by western blot. (**d**) FACS analysis of surface marker molecule, 50 μg exosomes was incubated with 100 μl of 4-μm-diameter aldehyde/sulfate latex beads and stained with CD63-PE antibody (blue line) or negative control antibody (red line). (**e**) Distribution of RNA sequence length over all sequence in exosomes. (**f**) MiRNA levels in exosomes of RAW 264.7 cells with or without RANKL induction for 2 days were analyzed by qRT-PCR. (**g**) Exosomes from RANKL-induced RAW 264.7 cells were isolated and incubated with the indicated reagents overnight at 4 °C. RNA was isolated from these exosomes and used for the analysis of miR-214 levels by qRT-PCR. (**h**) The cycle threshold (CT) value of miR-214 in AB (apoptotic body), MV (microvesicle) and exosome from the culture medium of RAW 264.7 cells were analyzed by qRT-PCR. (**i**) Relative tartrate-resistant acid phosphatase (TRAP) protein levels in human OC with and without M-CSF (25 ng ml^−1^) and RANKL (30 ng ml^−1^) treatment for 6 days. (**j**) Representative images of TRAP staining of human OC with and without M-CSF and RANKL treatment. (**k**) miR-214 level in exosomes of human OC treated with M-CSF and RANKL or not. (**l**) miR-214 level in exosomes of human OC. The exosomes were isolated and treated with RNase A or RNase A+Triton X-100. OC, osteoclast. The data represent the mean±s.e.m. of three independent experiments. *P<0.05 and **P<0.01.

To characterize the vesicles released during osteoclastogenesis, mouse osteoclast progenitor RAW 264.7 cells were induced by RANKL for 2 days. These cells showed a high expression of tartrate-resistant acid phosphatase by western blot and immunohistochemistry staining, which exhibited multinuclear osteoclasts (Supplementary Figure S1a and b). The medium was then collected to analyze the size distribution of particles by dynamic light scattering. Two peaks, with diameters of 12 and 75 nm, respectively, were identified (Figure 1a). The latter vesicles were isolated via a series of microfiltration and ultracentrifugation steps. Electron microscopy revealed vesicles ~50–100 nm in diameter with a rounded morphology, the typical shape of exosomes (Figure 1b). The results of western blot analysis confirmed the proteins expected to be present in the exosomes such as HSP70, TSG101 and the absence of nuclear proteins such as TFIIB and LaminA/C (Figure 1c). We also detected the existence of transmembrane protein CD63 in the exosome by fluorescence-activated cell sorting (Figure 1d). Next, we examined the distribution of RNA sequence length in exosomes. Bioanalyzer analysis revealed the enrichment of RNAs averaging 18–24 nucleotides (nt) in length, the typical length of miRNAs (Figure 1e). Deep sequence analysis showed 70 common miRNAs present in exosomes from RAW 264.7 cells and RANKL-induced RAW 264.7 cells. Twelve miRNAs were identified only in RAW 264.7 exosomes and three only in RANKL-induced RAW 264.7 exosomes (Supplementary Table S1). Among these miRNAs, 13 are reportedly involved in bone metabolism [36–54]. The levels of these 13 miRNAs were further confirmed by quantitative real-time polymerase chain reaction (qRT-PCR) analysis from the same amount of exosomes (Supplementary Figure S1c). Nine miRNAs (miR-148a-3p, miR-183a-5p, miR-214-3p, miR-27a-3p, miR-92a-3p, miR-378a-3p, miR-23a-3p, miR-21a-5p and miR-16-5p) were upregulated, and four (miR-155-5p, miR-199a-3p, miR-320-3p and miR-125a-5p) were downregulated in exosomes from RANKL-induced RAW 264.7 cells compared with RAW 264.7 cells (Figure 1f and Supplementary Figure S1d). Among the upregulated miRNAs, miR-214 has been demonstrated to be upregulated during osteoclastogenesis and inhibit osteoblast function by targeting *ATF4* [42, 55], which hints that miR-214 in the exosome may be involved in the crosstalk between osteoclasts and osteoblasts. miR-214 in lipid-bilayered exosomes was protected from RNase degradation (Figure 1g). Quantitative analysis of miR-214 was performed on the pellet of extracellular vesicles generated by differential centrifugation. According to the CT value of miR-214, we found that miR-214 mainly existed in exosome but not in AB (apoptotic body) and MV (microvesicle) isolated from RAW 264.7 cell culture medium (Figure 1h). To further verify these results, human CD14^+^ peripheral blood mononuclear cells were isolated, and osteoclastogenesis was induced by macrophage colony-stimulating factor (M-CSF) and RANKL (Figure 1i and j). Similarly, miR-214 levels were increased in exosomes during the process of osteoclastogenesis (Figure 1k) and protected from RNase degradation (Figure 1l). These results suggest that osteoclast secretes miRNA-contained exosomes, which include miR-214, the key regulator of bone remodeling.

### Osteoclast-derived exosomes transfer miR-214 to osteoblasts and inhibit their activity

**Figure 2:**
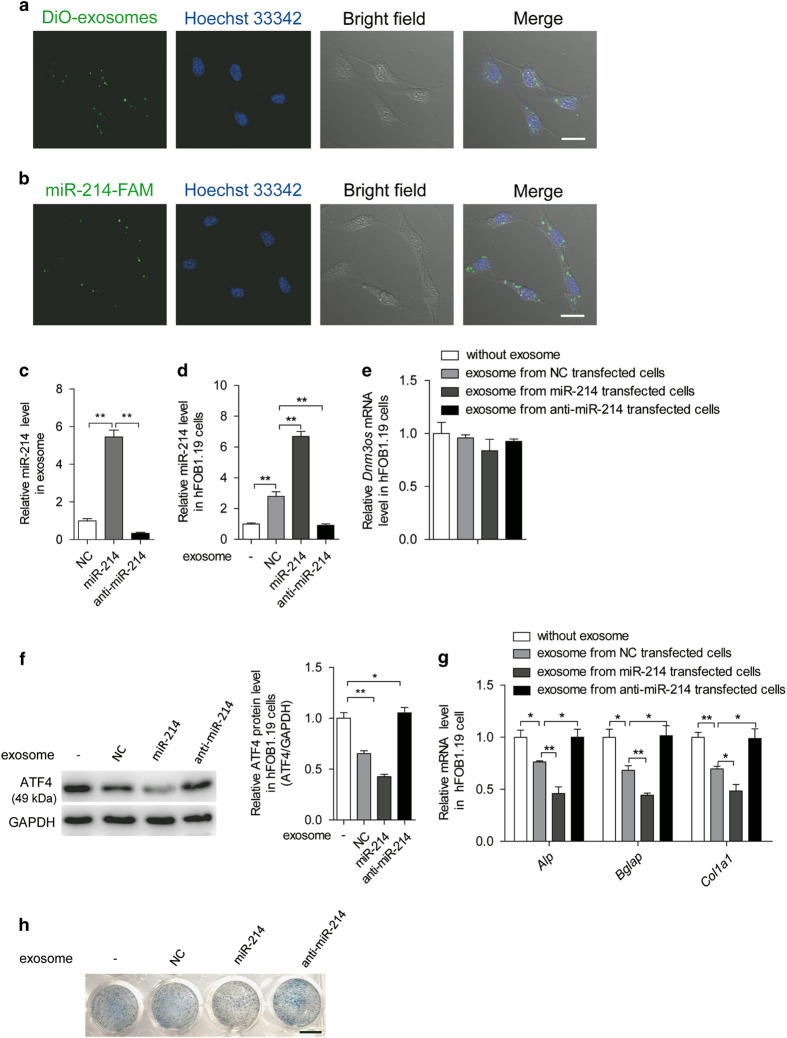
Osteoclasts transmit miR-214 to osteoblasts and regulate their activity. (**a**) Confocal microscopy images of colocalization of exosomes from RANKL-induced RAW 264.7 cells with MC3T3-E1 cells. Exosomes were labeled by 3′-dioctadecyloxacarbocyanine perchlorate (DiO) (green) and cell nuclei were stained with Hoechst 33342 (blue). Scale bars, 25 μm. (**b**) Confocal microscopy images of colocalization of miR-214 in exosomes with MC3T3-E1 cells. miR-214 in exosomes from RANKL-induced RAW 264.7 cells were labeled by FAM (green) and cell nuclei were stained with Hoechst 33342 (blue). Scale bars, 25 μm. (**c**) qRT-PCR analysis of miR-214 levels in exosomes isolated from human osteoclasts transfected with NC (negative control), miR-214 mimics (miR-214) or its inhibitor (anti-miR-214) under the treatment of M-CSF (25 ng ml^−1^) and RANKL (30 ng ml^−1^) for 6 days. miR-214 levels were normalized to *RNU6*. (**d**) miR-214 level in hFOB1.19 cells was analyzed by qRT-PCR after incubation with the same number of exosomes (5 mg ml^−1^) from human osteoclasts transfected without or with NC, miR-214 or anti-miR-214 for 2 days. (**e**) The change of *Dnm3os* mRNA level in hFOB1.19 cells after incubation with exosomes from OC transfected with NC, miR-214 or anti-miR-214 for 2 days. *Dnm3os* mRNA level was analyzed by qRT-PCR and normalized to *Gapdh*. (**f**) Expression of ATF4 protein in hFOB1.19 cells after incubation with the same number of exosomes from human osteoclasts transfected with or without NC, miR-214 or anti-miR-214 for 2 days. (**g**) *Alp*, *Bglap* and *Col1α1* mRNA levels in hFOB1.19 cells were analyzed by qRT-PCR after incubation with the same number of exosomes from human osteoclasts transfected without or with NC, miR-214 or anti-miR-214 for 2 days, and the transcript levels were normalized to *Gapdh*. (**h**) Representative images of Alp staining of hFOB1.19 cells after incubation with the same number of exosomes from human osteoclasts transfected without or with NC, miR-214 and anti-miR-214 for 6 days. The culture medium replaced for fresh medium every 2 days. Scale bar, 7.8 mm. The data represent the mean±s.e.m. of three independent experiments. *P<0.05 and **P<0.01.

To assess whether exosomes from RANKL-induced mouse osteoclast cells can be internalized by mouse preosteoblast MC3T3-E1 cells, exosomes were labeled with 3′-dioctadecyloxacarbocyanine perchlorate (green). Labeled exosomes were incubated with MC3T3-E1 cells for 60 min at 37 °C. Cells were then observed by confocal microscopy, which revealed the incorporation of exosomes into MC3T3-E1 cells (Figure 2a). Next, we examined whether miR-214 is transferred via exosomes from osteoclasts to osteoblasts. When MC3T3-E1 cells were cultured in the presence of exosomes collected from the supernatant of osteoclasts transfected with FAM-labeled miR-214, the cells exhibited a fine granular fluorescent pattern within the cytoplasm, indicating the incorporation of miR-214 into MC3T3-E1 cells (Figure 2b).

To further explore the role of miR-214 in exosome function in human osteoblast hFOB1.19 cells, exosomes were isolated from the supernatant of RANKL-induced human osteoclasts transfected with miR-214 mimics, anti-miR-214 or negative control (NC). miR-214 levels and copies in the exosomes were markedly upregulated by miR-214 mimics and downregulated by anti-miR-214 (Figure 2c and Supplementary Figure S2a). When those exosomes were incubated with hFOB1.19 cells, miR-214 levels and copies in these cells were changed accordingly with the levels in the exosomes (Figure 2d and Supplementary Figure S2b). However, there was no change in mRNA levels of *Dnm3os*, which encodes miR-214 (Figure 2e), suggesting that miR-214 was transferred from the exosomes to hFOB1.19 cells. Consistent with these results, the expression of Atf4 protein, the target of miR-214 in osteoblasts, was also altered by exosomes harboring different miR-214 levels (Figure 2f). Functionally, *Alp*,* Bglap* and *Col1α1* mRNA levels were significantly reduced in hFOB1.19 cells by exosomes from miR-214 mimic-transfected human osteoclasts compared with the NC and anti-miR-214 treatment groups (Figure 2g). Consistent with the changes in *Alp* mRNA levels, cells that received exosomes with lower miR-214 levels displayed enhanced Alp staining, whereas those with higher miR-214 levels exhibited weak Alp staining (Figure 2h). The results using mouse osteoclasts were similar to those of human osteoclasts (Supplementary Figure S2c–g).

To further confirm the role of miR-214 in this process, we antagonized miR-214 level in osteoblasts with anti-miR-214. When these osteoblasts were incubated with exosomes, there were no changes of miR-214 levels in these cells. At the same time, Atf4 protein levels as well as *Alp*, *Bglap* and *Col1α1* mRNA levels in these osteoblast remained unchanged (Supplementary Figure S2h–j). These results demonstrated that miR-214 is essential in the exosome-mediated inhibitory effect on osteoblasts.

Next, a coculture assay system, in which MC3T3-E1 cells were cocultured with RANKL-induced RAW 264.7 cells, was used to assess miR-214-mediated crosstalk between osteoblasts and osteoclasts (Supplementary Figure S2k). miR-214 levels were markedly increased when MC3T3-E1 cells were incubated with miR-214 mimic-transfected RAW 264.7 cells (Supplementary Figure S2l). Moreover, levels of ATF4 protein and *Alp*, *Bglap* and *Col1α1* mRNAs were decreased (Supplementary Figure S2m and n) in MC3T3-E1 cells relative to those in cells cocultured with NC RAW 264.7 cells. However, there was no change in mRNA levels of *Dnm3os*, which encodes miR-214 (Supplementary Figure S2o).

To further monitor the transport of miR-214 from osteoclasts to osteoblasts, we designed a miR-214 star variant (miR-214*) (Supplementary Figure S2p), which could be distinguished from miR-214 by quantitative reverse transcription-PCR (qRT-PCR) (Supplementary Figure S2q), and the absolute copies of miR-214* and miR-214 in exosomes and osteoblasts could be quantified. The results showed that miR-214* copies in the exosomes secreted from miR-214*-transfected mouse osteoclasts were eight times of miR-214 copies (Supplementary Figure S2r). When MC3T3-E1 cells were incubated with these exosomes, the miR-214* copies in these cells were three times of miR-214 levels (Supplementary Figure S2s). When MC3T3-E1 cells were cocultured with mouse osteoclasts, miR-214* copies in MC3T3-E1 cells were about 2.5 times of miR-214 levels in MC3T3-E1 cells (Supplementary Figure S2t). Taken together, these findings indicate that secreted miR-214-containing exosomes from osteoclasts can be taken up by osteoblasts, resulting in the transfer of miR-214 into osteoblasts and thus downregulation of miR-214 target gene expression.

### Inhibition of exosome formation and secretion from osteoclasts attenuates the effect of osteoclasts on osteoblasts

**Figure 3:**
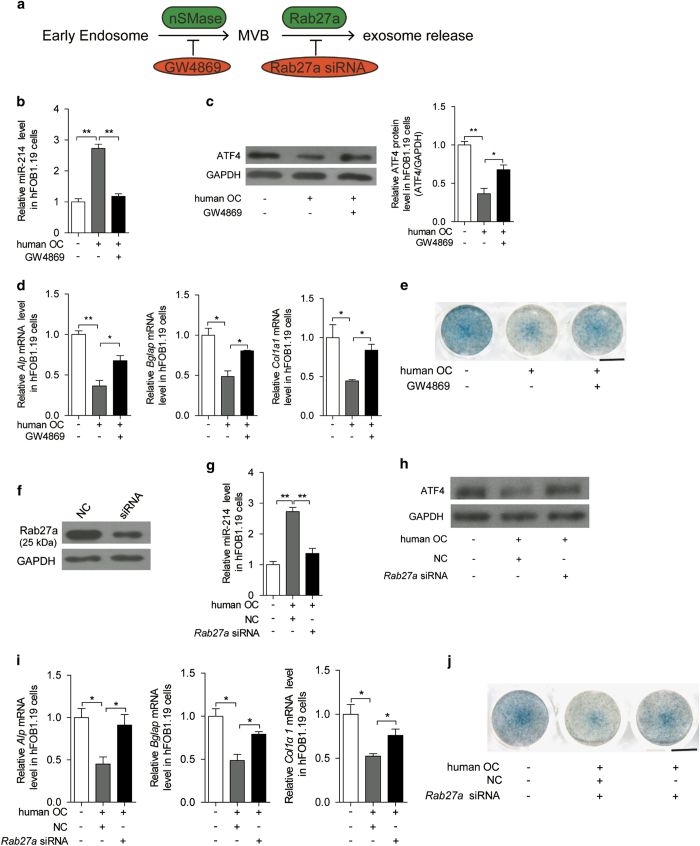
Inhibition of exosome release impairs the miR-214-mediated inhibitory effect of osteoclasts on osteoblasts. (**a**) Inhibition of exosome secretion by specific targeting neutral sphingomyelinase (nSMase) and Rab27a. (**b**) miR-214 levels were analyzed by qRT-PCR in hFOB1.19 cells, which were cocultured for 2 days with human OC with or without the N-SMase inhibitor GW4869 treatment (20 μm). (**c**) Expression of ATF4 protein in hFOB1.19 cells, which were cocultured for 2 days with human OC with or without GW4869 treatment (20 μm) were analyzed by western blot and were normalized to GAPDH. (**d**) The mRNA levels of *Alp*, *Bglap* and *Col1α1* were analyzed by qRT-PCR in hFOB1.19 cells, which were cocultured with human OC with or without GW4869 treatment. (**e**) Representative images of Alp staining of hFOB1.19 cells, which were cocultured for 6 days with RANKL-induced human OC cells with or without GW4869 treatment (20 μm). The culture medium replaced for fresh medium every 2 days. Scale bar, 7.8 mm. (**f**) Expression of Rab27a protein were analyzed by western blot and normalized to GAPDH in human OC transfected with NC and *Rab27a* siRNA for 2 days. (**g**) miR-214 level was analyzed by qRT-PCR in hFOB1.19 cells, which were cocultured with human OC transfected with NC and *Rab27a* siRNA for 2 days. (**h**) Expression of ATF4 protein in hFOB1.19 cells, which were cocultured with human OC transfected with NC and *Rab27a* siRNA was analyzed by western blot and were normalized to GAPDH. (**i**) *Alp*, *Bglap* and *Col1α1* mRNA levels were analyzed by qRT-PCR in hFOB1.19 cells, which were cocultured with human OC transfected with NC and *Rab27a* siRNA. (**j**) Representative images of Alp staining of hFOB1.19 cells, which were cocultured with RANKL-induced human OC cells transfected with NC and *Rab27a* siRNA for 6 days. The culture medium replaced for fresh medium every 2 days. Scale bar, 7.8 mm. OC, osteoclasts. The data represent the mean±s.e.m. of three independent experiments. *P<0.05 and **P<0.01.

To investigate the role of exosomes in the transfer of miR-214, exosome production was blocked by the neutral sphingomyelinase inhibitor GW4869 [56], and exosome release inhibited by *Rab27a* small interfering RNA (siRNA) (Figure 3a and Supplementary Figure S3a and b), GW4869 and *Rab27a* siRNA had no apparent direct effect on miR-214 level or osteoclast activity (Supplementary Figure S3c–f). miR-214 transfer was attenuated when exosome production in osteoclasts was inhibited by GW4869 (Figure 3b). Accordingly, GW4869 reversed miR-214 transmission-induced downregulation of ATF4 protein levels and *Alp*, *Bglap* and *Col1α1* mRNA levels in hFOB1.19 cells (Figure 3c and d). Alp staining showed similar changes (Figure 3e). Moreover, when exosome release from osteoclasts was inhibited by transfection of *Rab27a* siRNA (Figure 3f), miR-214 transfer from osteoclasts to osteoblasts was also attenuated (Figure 3g). Accordingly, *Rab27a* knockdown reduced miR-214 transmission-induced downregulation of Atf4 protein levels, in addition to *Alp*, *Bglap* and *Col1α1* mRNA levels and Alp staining in hFOB1.19 cells (Figure 3h–j). The transfer of miR-214 from mouse osteoclasts to mouse osteoblasts was inhibited by GW4869 or *Rab27a* siRNA in a similar manner (Supplementary Figure S3g–o). These results suggest that both the transfer of miR-214 from osteoclasts and its function in osteoblasts are dependent on exosomes.

### Osteoclast-derived exosomes recognize osteoblasts via ephrinA2/EphA2

**Figure 4:**
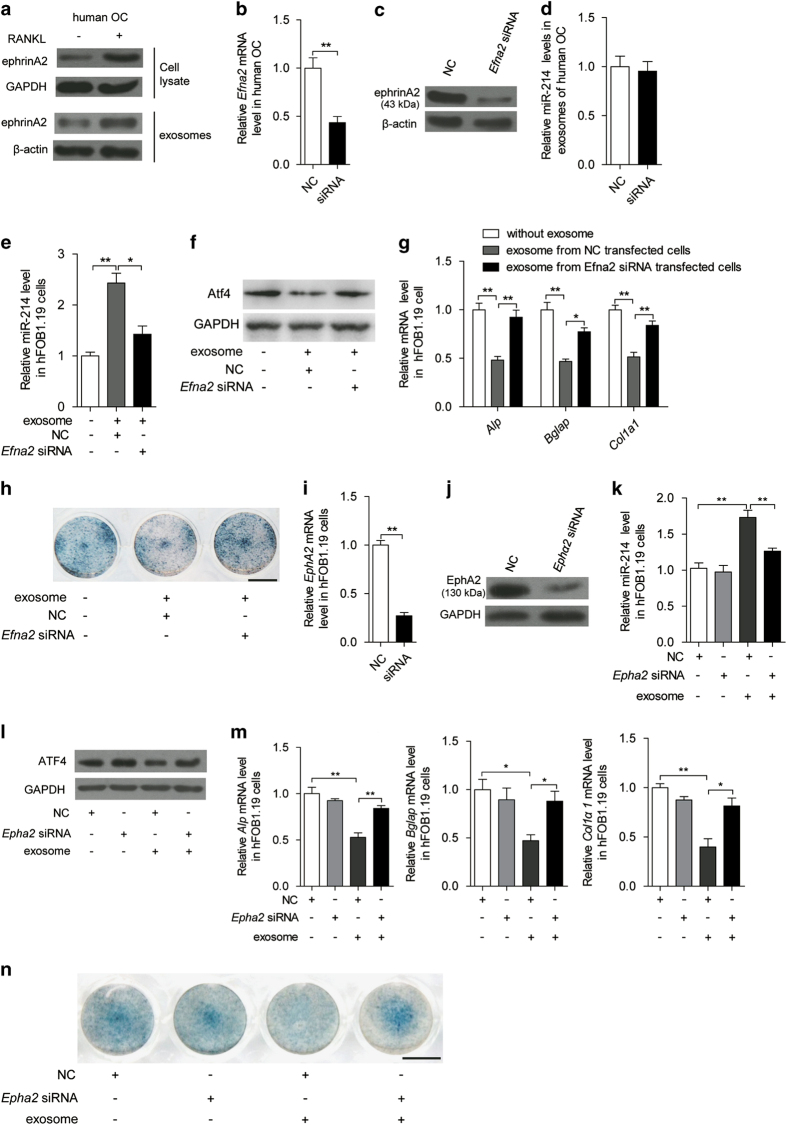
EphrinA2 and EphA2 are required for the function of exosomes on osteoblasts. (**a**) The protein levels of ephrinA2 in cell lysate and exosomes from human CD14^+ ^PBMCs with M-CSF (25 ng ml^−1^) and RANKL (30 ng ml^−1^) induction or not. EphrinA2 protein levels were normalized to GAPDH or β-actin. (**b**) *Efna2* mRNA level in *Efna2* siRNA-transfected human OC were analyzed by qRT-PCR. (**c**) The change of ephrinA2 protein level in exosomes from *Efnα2* siRNA-transfected human OC. EphrinA2 protein levels were normalized to β-actin. (**d**) The change of miR-214 levels in exosomes from *Efna2* siRNA-transfected human OC were analyzed by qRT-PCR. (**e**) miR-214 levels in hFOB1.19 cells were analyzed after incubation for 2 days without or with exosomes collected from the same number of *Efna2* siRNA-transfected human OC by qRT-PCR. (**f**) The change of ATF4 protein level in hFOB1.19 cells after incubation without or with the same number of exosomes collected from human OC or *Efnα2* siRNA-transfected human OC, ATF4 levels were analyzed by western blot and were normalized to GAPDH. (**g**) The changes of *Alp*, *Bglap* and *Col1α1* mRNA levels in hFOB1.19 cells were analyzed after incubation without or with exosomes from *Efna2* siRNA-transfected human OC by qRT-PCR. (**h**) Representative images of Alp staining of hFOB1.19 cells after incubation without or with exosomes from *Efnα2* siRNA-transfected human OC cells for 6 days. Scale bar, 7.8 mm. (**i**) *Epha2* mRNA levels in *Epha2* siRNA-transfected hFOB1.19 cells were analyzed by qRT-PCR. (**j**) The change of EphA2 protein level in hFOB1.19 cells was analyzed by western blot. EphA2 protein levels were normalized to GAPDH. (**k**) miR-214 levels in hFOB1.19 cells transfected with *Epha2* siRNA were analyzed by qRT-PCR after incubation with exosomes from human OC. (**l**) The changes of ATF4 protein level in hFOB1.19 cells after incubation with exosome from human OC. hFOB1.19 cells were transfected with *Epha2* siRNA or not. (**m**) *Alp*, *Bglap* and *Col1α1* mRNA levels in *Epha2* siRNA-transfected hFOB1.19 cells after incubation with exosome from human OC were analyzed by qRT-PCR. The mRNA levels were normalized to *Gapdh*. (**n**) Representative images of Alp staining of *Ephα2* siRNA-transfected hFOB1.19 cells after incubation with exosome from RANKL-induced human OC cells for 6 days. Scale bar, 7.8 mm. OC, osteoclast. The data represent the mean±s.e.m. of three independent experiments. *P<0.05 and **P<0.01.

In general, exosomes express cell recognition molecules on their surfaces that facilitate selective targeting and uptake by recipient cells. In this regard, osteoclast-derived, miR-214-containing exosomes act as a coupling inhibitor to negatively regulate osteoblasts. Among the coupling inhibitors, osteoclast-derived ephrinA2, whether anchored onto osteoclast membranes or in soluble form, acts on osteoblasts through its Eph receptor [57]. To investigate whether this interaction is involved in the miR-214-containing exosome recognition of osteoblasts, ephrinA2 protein levels were examined in human osteoclasts and exosomes. EphrinA2 protein was enriched in cell lysates and exosomes (Figure 4a). When ephrinA2 was knocked down in human osteoclasts by *Efna2* siRNA (Figure 4b and c), miR-214 levels remained unchanged in the exosomes derived from these human osteoclasts (Figure 4d). However, when hFOB1.19 cells were incubated with exosomes isolated from the same number of human osteoclasts transfected with *Efna2* siRNA, miR-214 levels were significantly attenuated compared with the NC (Figure 4e). Consistent with these results, Atf4 protein levels, as well as *Alp*, *Bglap* and *Col1α1* mRNA levels and Alp staining, were also increased in these hFOB1.19 cells (Figure 4f–h).

EphA2 is the ephrinA2 receptor on osteoblasts. To investigate the role of EphA2 in the recognition of osteoclast-derived exosomes by osteoblasts, *Epha2* was knocked down in hFOB1.19 cells using *Epha2* siRNA (Figure 4i and j). miR-214 levels were not upregulated by exosomes from human osteoclasts in hFOB1.19 cells transfected with *Epha2* siRNA (Figure 4k). Accordingly, Atf4 protein levels, as well as *Alp*, *Bglap* and *Col1α1* mRNA levels and Alp staining, were not decreased in hFOB1.19 cells transfected with *Epha2* siRNA when incubated in the presence of these exosomes (Figure 4l–n). Results in mouse osteoclasts and osteoblasts were similar to those in human osteoclasts and osteoblasts (Supplementary Figure S4a–n). To investigate whether downstream pathway of EphA2 in osteoblasts was activated after incubation with ephrinA2-containing exosomes, we compared the changes of RhoA activity in osteoblasts after incubation with exosomes derived from osteoclasts transfected with Efna2 siRNA or control. The results did not show any difference between them (Supplementary Figure S4o).

Another previously identified coupling inhibitor, in addition to ephrinA2, is semaphorin 4D (Sema4D), which is expressed in osteoclasts. Sema4D binds to its receptor, Plexin-B1, in osteoblasts to inhibit bone formation [9, 11]. However, Sema4D knockdown in osteoclasts had no effect on osteoclast-derived exosome-mediated miR-214 transfer from osteoclasts to osteoblasts (Supplementary Figure S5a–e). These results suggest that ephrinA2 is a marker used by osteoclast-derived exosomes to recognize osteoblasts, and the ephrinA2/EphA2 interaction is essential for exosome-mediated crosstalk between osteoclasts and osteoblasts.

### Exosomes from osteoclast-specific miR-214-overexpressing mice inhibit osteoblast function *in vivo*

**Figure 5:**
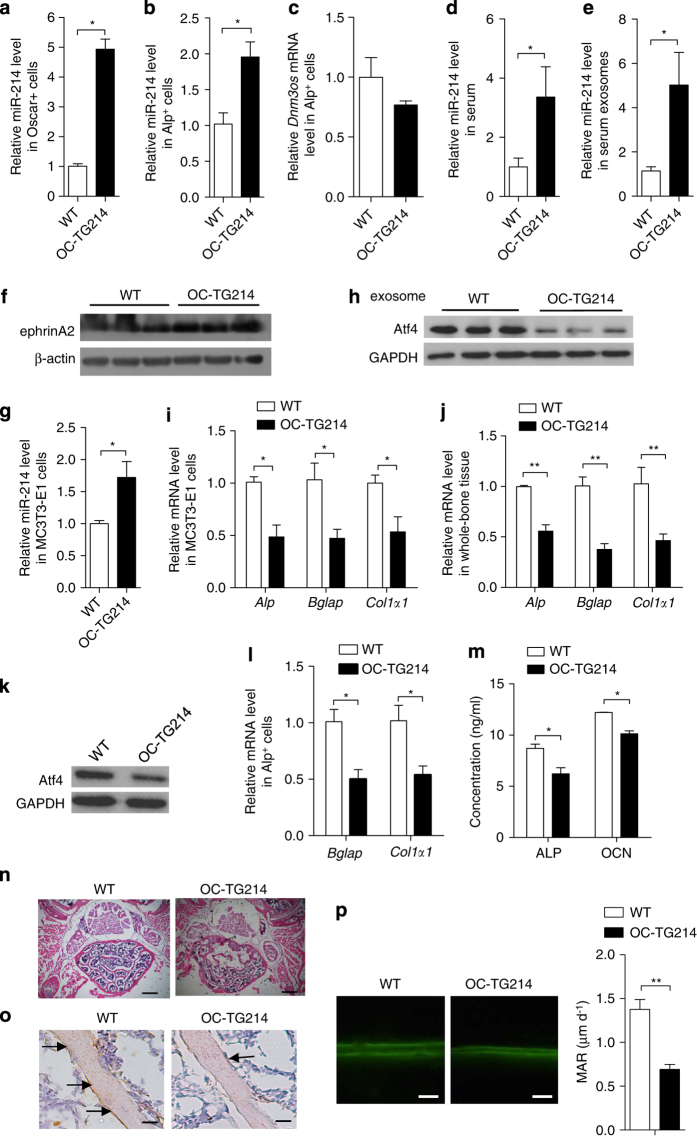
Osteoclast-specific miR-214 transgenic mice secrete exosomes enriched in miR-214 to inhibit osteoblast activity *in vivo*. (**a** and **b**) qRT-PCR analysis of miR-214 levels in Oscar^+^ cells and Alp^+^ cell separated by cell sorting with flow cytometry from WT and OC-TG214 mice. miR-214 levels were normalized to *RNU6*. (**c**) qRT-PCR analysis of *Dnm3os* levels in Alp^+^ cell separated by cell sorting with flow cytometry from WT and OC-TG214 mice. *Dnm3os* levels were normalized to *Gapdh*. (**d**) qRT-PCR analysis of miR-214 levels in serum (normalized to cel-miR-39) from WT and OC-TG214 mice. WT, *n*=5; OC-TG214, *n*=6. (**e**) qRT-PCR analysis of miR-214 levels in exosomes (normalized to RNU6) from the serum of WT and OC-TG214 mice. WT, *n*=5; OC-TG214, *n*=6. (**f**) Western blot analysis of ephrinA2 protein levels in serum exosomes from OC-TG214 and WT mice. EphrinA2 protein levels were normalized to β-actin. (**g**) qRT-PCR analysis of miR-214 level in MC3T3-E1 cells after incubation with exosomes from serum of WT and OC-TG214 mice. (**h**) The change of Atf4 protein level in MC3T3-E1 cells after incubation with exosome from serum of WT and OC-TG214 mice for 2 days, Atf4 levels were analyzed by western blot and were normalized to GAPDH. (**i**) qRT-PCR analysis of *Alp*, *Bglap* and *Col1α1* mRNA level in MC3T3-E1 cells after incubation with exosomes from serum of WT and OC-TG214 mice for 2 days. The PCR products were normalized to *Gapdh*. (**j**) qRT-PCR analysis of *Alp*, *Bglap* and *Col1α1* mRNA in whole-bone tissue of WT and OC-TG214 mice. (**k**) Western blot analysis of Atf4 protein levels in whole-bone tissue of WT and OC-TG214 mice, Atf4 levels were analyzed by western blot and were normalized to GAPDH. (**l**) qRT-PCR analysis of *Alp*, *Bglap* and *Col1α1* mRNA in Alp^+^ cells of WT and OC-TG214 mice, the PCR products were normalized to *Gapdh*. (**m**) ELISA analysis of serum ALP, OCN levels in WT and OC-TG214 mice. (**n**) Images for hematoxylin and eosin (HE) staining in the fifth lumbar vertebral body of WT and OC-TG214 mice. Scale bars, 1000 μm. (**o**) Histology images for Alp staining in the fifth lumbar vertebral body of WT and OC-TG214 mice. Arrows indicate the number of osteoblast, scale bars, 100 μm. (**p**) Representative images of new bone formation assessed by double calcein labeling. MAR, mineral apposition rate. Scale bars, 20 μm. The data represent the mean±s.e.m. of three independent experiments. *P<0.05 and **P<0.01.

To investigate the role of osteoclast-derived miR-214 in the suppression of osteoblast function *in vivo*, we used osteoclast-specific *Acp5* miR-214 transgenic (OC-TG214) mice [55] and found that miR-214 levels were markedly higher in osteoclasts (Oscar^+^ cells) isolated from OC-TG214 mice (Figure 5a). qRT-PCR analysis of whole femurs showed that *Nfatc1*, *Acp5*, *Mmp9*, *Clcn7* and *Ctsk* mRNA levels were significantly higher in OC-TG214 mice compared with wild-type (WT) mice (Supplementary Figure S6a). Serum levels of bone resorption markers C-telopeptide of type I collagen and N-telopeptide of type I collagen were also increased in OC-TG214 mice (Supplementary Figure S6b). miR-214 level in osteoblasts (Alp^+^ cells) isolated from OC-TG214 mice was also significantly upregulated (Figure 5b). However, *Dnm3os* mRNA levels remain unchanged (Figure 5c), which indicated that the increased miR-214 in osteoblasts was mainly from its importation from osteoclasts. Interestingly, circulatory miR-214 levels both in the serum and exosomes isolated from the serum were substantially increased in OC-TG214 mice compared with WT mice (Figure 5d and e). The marker protein of osteoclast-derived exosomes, ephrinA2, was also increased in exosomes from the serum of OC-TG214 compared with WT mice (Figure 5f). To analyze the effect of miR-214-containing exosomes from OC-TG214 mice on osteoblast function, serum exosomes were isolated and incubated with MC3T3-E1 cells. miR-214 levels were significantly increased in MC3T3-E1 cells following incubation with exosomes from the serum of OC-TG214 mice (Figure 5g). Accordingly, Atf4 protein levels, as well as *Alp*, *Bglap* and *Col1α1* mRNA levels were markedly reduced compared with those in cells incubated with exosomes from WT mice (Figure 5h and i). Atf4 protein and *Alp*, *Bglap* and *Col1α1* mRNA levels in whole-bone tissue and Alp^+^ osteoblasts from OC-TG214 mice were decreased markedly *in vivo* (Figure 5j–l). Additionally, serum ALP and OCN levels were much lower compared with those from WT mice (Figure 5m). To further evaluate the effect of osteoclast-specific miR-214 overexpression on osteoblasts *in vivo*, hematoxylin staining on the fifth lumbar vertebral body was performed, which showed less trabecular number and lower bone mineral density in OC-TG214 mice compared with that in WT mice (Figure 5n). Histology images for Alp staining showed decreased Alp level in bone surface of OC-TG214 mice compared with that in WT mice (Figure 5o). A dynamic bone histomorphometric analysis of the distal femurs was performed, which showed that the bone formation rate was significantly lower in OC-TG214 mice compared with in WT mice (Figure 5p). However, osteoclast activity in osteoblast-specific transgenic miR-214 mice (OB-TG214) remained similar to that in WT mice (Supplementary Figure S6c–f), suggesting that miR-214 may be unidirectionally transferred from osteoclasts to osteoblasts *in vivo*.

### Characteristics of exosomes from osteoporotic patients and mice and the inhibitory effect of miR-214 on osteoblasts

**Figure 6:**
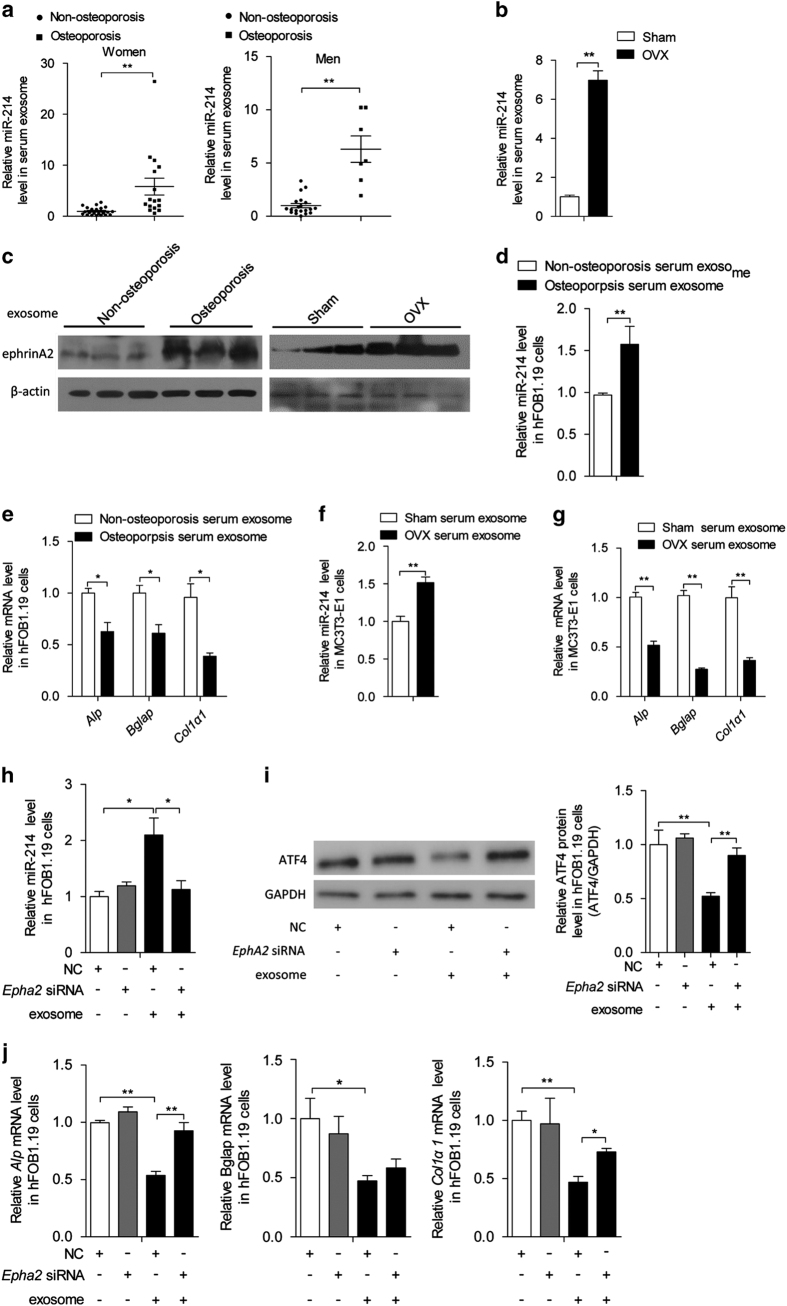
Changes in circulating miR-214 levels in exosomes from osteoporotic patients and OVX mice and the inhibitory role of exosomes on osteoblast activity. (**a**) qRT-PCR analysis of circulating miR-214 in serum exosomes (normalized to *RNU6*) from osteoporosis vs non-osteoporosis people. Non-osteoporosis women, *n*=22; non-osteoporosis men, *n*=16; osteoporosis women, *n*=20; osteoporosis men, *n*=7. (**b**) qRT-PCR analysis of miR-214 levels in serum exosomes (normalized to RNU6) of sham and OVX mice. Sham, *n*=6; OVX, *n*=6. (**c**) Western blot analysis of ephrinA2 protein levels in serum exosomes from osteoporosis and non-osteoporosis patients, or OVX and sham mice. (**d**) miR-214 level in hFOB1.19 cells cultured in 12-well plate was analyzed by qRT-PCR after incubation with exosomes from 2 ml serum of patients for 2 days. miR-214 levels were normalized to *RNU6*. (**e**) *Alp*, *Bglap* and *Col1α1* mRNA levels in hFOB1.19 cells were analyzed by qRT-PCR after incubation with exosomes from the serum of patients. (**f**) miR-214 level in MC3T3-E1 cells cultured in 24-well plate was analyzed by qRT-PCR after incubation with exosomes from 1 ml serum (pooled from four mice) of OVX or sham mice for 2 days. miR-214 levels were normalized to *RNU6*. (**g**) *Alp*, *Bglap* and *Col1α1* mRNA levels in MC3T3-E1 cells were analyzed by qRT-PCR after incubation with exosomes from the serum of OVX or sham mice for 2 days. (**h**) miR-214 levels in *Epha2* siRNA-transfected hFOB1.19 cells after incubation for 2 days with 2 ml serum exosomes from osteoporosis people were analyzed by qRT-PCR. (**i**) The change of ATF4 protein level in *Epha2* siRNA-transfected hFOB1.19 cells after incubation with serum exosomes from osteoporosis people, ATF4 levels were analyzed by western blot and were normalized to GAPDH. (**j**) *Alp*, *Bglap* and *Col1α1* mRNA levels in *Epha2* siRNA-transfected hFOB1.19 cells after incubation with serum exosomes from osteoporosis people were analyzed by qRT-PCR. The data represent the mean±s.e.m. of three independent experiments. *P<0.05 and **P<0.01.

To investigate the diagnostic potential of miR-214, as a secreted biomarker, in osteoporosis, miR-214 levels were analyzed in osteoporotic patients and mice. First, the levels of miR-214 in the serum from osteoporotic patients and non-osteoporotic individuals were determined. A significant difference between the two populations was observed in both men and women (Supplementary Table S2 and Supplementary Figure S7a). Next, miR-214 levels in the serum from OVX and sham mice were examined, which revealed that miR-214 levels were substantially increased in OVX mice compared with the sham mice (Supplementary Figure S7b). miR-214 levels in Oscar^+^ cells and Alp^+^ cells were markedly upregulated in OVX mice (Supplementary Figure S7c). However, the mRNA levels of *Dnm3os* were only increased in Oscar^+^ cells but unchanged in Alp^+^ cells (Supplementary Figure S7d). Accordingly, *Nfatc1*, *Acp5*, *Mmp9*, *Clcn7* and *Ctsk* mRNA levels were upregulated in whole-bone tissues (Supplementary Figure S7e), and serum C-telopeptide of type I collagen and N-telopeptide of type I collagen levels were increased (Supplementary Figure S7f). In contrast, ALP and OCN protein levels were decreased in the serum (Supplementary Figure S7g). Next, exosomes were isolated from the serum and measured for miR-214 levels. qRT-PCR revealed that miR-214 levels in the exosomes from patient sera and OVX mice were markedly higher compared with those of their respective controls (Figure 6a and b). Similar results were also observed in hindlimb suspension-induced bone loss mice (Supplementary Figure S8a–e). EphrinA2 levels were also analyzed, which were much higher in serum exosomes of osteoporotic patients and OVX mice (Figure 6c). To further investigate the clinical significance of miR-214 carried by exosomes, as a circulating miRNA, in the inhibition of bone formation, exosomes from the serum of osteoporotic patients and non-osteoporotic individuals were isolated and incubated with osteoblasts. miR-214 levels in hFOB1.19 cells were much higher when incubated with exosomes from osteoporotic patients than non-osteoporotic individuals (Figure 6d). Consistently, *Alp*, *Bglap* and* Col1α1* mRNA levels were markedly reduced in hFOB1.19 cells (Figure 6e). A similar role of exosomes from OVX mice in the regulation of MC3T3-E1 cells was also observed (Figure 6f and g). To confirm the role of EphA2 in the recognition of human osteoclast-derived exosomes by osteoblasts, *Epha2* was knocked down in hFOB1.19 cells using *Epha2* siRNA. miR-214 levels in these cells were not upregulated by exosomes from osteoporotic patients (Figure 6h). Accordingly, ATF4 protein levels and *Alp*, *Bglap* and *Col1α1* mRNA levels in hFOB1.19 cells transfected with *Epha2* siRNA were not reduced following incubation with these exosomes (Figure 6i and j). These findings underscore the clinical significance of elevated miR-214 levels in the serum of osteoporotic patients and suggest its potential use as a marker for the diagnosis of osteoporosis and as a target for osteoporosis therapeutics.

### Inhibition of exosome release from OVX mice promotes osteoblast activity *in vivo*

**Figure 7:**
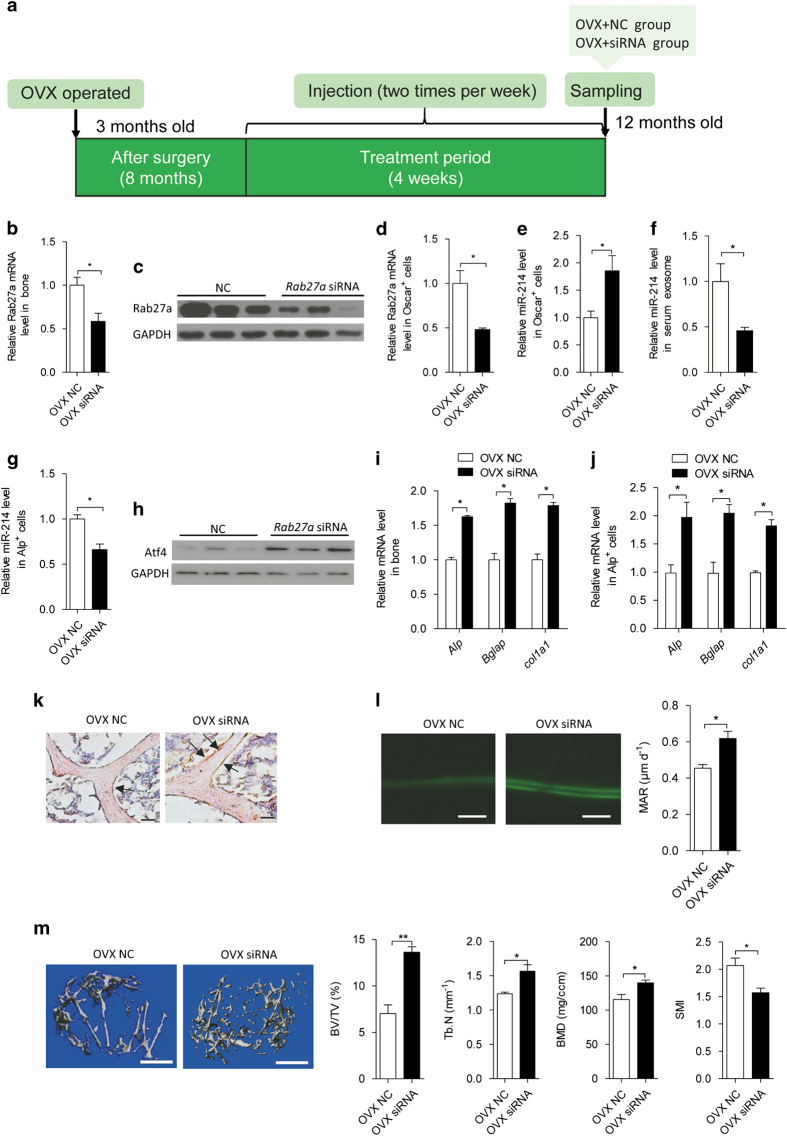
Blockade of exosome release protects against OVX-induced downregulated osteoblast activity *in vivo*. (**a**) A schematic diagram illustrating the experimental design. OVX NC, OVX mice injected with Rab27a siRNA-negtive control; OVX+siRNA, OVX mice injected with Rab27a siRNA. (**b**) qRT-PCR analysis of Rab27a mRNA levels in whole-bone tissue from OVX NC and OVX siRNA mice. OVX NC, *n*=4; OVX siRNA, *n*=4. The PCR products were normalized to *Gapdh*. (**c**) Western blot analysis of Rab27a protein levels in whole-bone tissue from OVX NC and OVX siRNA mice. (**d**) qRT-PCR analysis of Rab27a mRNA levels in Oscar^+^ cells separated by cell sorting with flow cytometry from OVX NC and OVX siRNA mice. (**e**) qRT-PCR analysis of miR-214 levels in Oscar^+^ cells separated from OVX NC and OVX siRNA mice. miR-214 levels were normalized to *RNU6*. (**f**) qRT-PCR analysis of miR-214 levels in Alp^+^ cells separated from OVX NC and OVX siRNA mice. miR-214 levels were normalized to *RNU6*. (**g**) qRT-PCR analysis of miR-214 levels in serum exosome from OVX NC and OVX siRNA mice. (**h**) Western blot analysis of Atf4 protein levels in whole-bone tissue from OVX NC and OVX siRNA mice. (**i** and **j**) qRT-PCR analysis of *Alp*, *Bglap* and *Col1α1* mRNA levels in whole-bone tissue and Alp^+^ cells of OVX NC and OVX siRNA mice. (**k**) Histology images for Alp staining in the fifth lumbar vertebral body of OVX NC and OVX siRNA mice. Arrows indicate the number of osteoblast. Scale bars, 100 μm. (**l**) Representative images of new bone formation assessed by double calcein labeling in the distal femurs. Scale bars, 20 μm. (**m**) Representative images showing three-dimensional trabecular architecture by micro-computed tomography (micro-CT) reconstruction in the distal femurs. BMD, bone mineral density; BV/TV, bone volume/tissue volume ratio; SMI, structure model index; Tb. N, trabecular number. Bars, 1 mm. The data represent the mean±s.e.m. of three independent experiments. *P<0.05 and **P<0.01.

To evaluate whether inhibition of exosome release from OVX mice prevents exosome-mediated inhibition of osteoblast function, pulsed systemic administration of *Rab27a* siRNA encapsulated with lipid nanoparticles was performed in estrogen-depleted mice 8 months after ovariectomy (Figure 7a). After injection with *Rab27a* siRNA, decreased *Rab27a* levels were confirmed in bone tissue and Oscar^+^ cells (Figure 7b–d). miR-214 levels in Oscar^+^ cells were significantly upregulated in *Rab27a* siRNA-treated mice (Figure 7e). However, miR-214 levels in serum, exosomes and osteoblasts were markedly reduced in *Rab27a* siRNA-treated mice (Figure 7f and g and Supplementary Figure S9a). Atf4 protein levels were increased in bone tissue (Figure 7h). Accordingly, *Alp*, *Bglap* and *Col1α1* mRNA levels in bone and Alp^+^ cells were increased (Figure 7i and j). In the serum from *Rab27a* siRNA-treated OVX mice, N-telopeptide of type I collagen levels were increased (Supplementary Figure S9b), as was ALP and OCN (Supplementary Figure S9c). qRT-PCR performed in whole femurs and Oscar^+^ cells showed that *Nfatc1*, *Acp5*, *Mmp9*, *Clcn7* and *Ctsk* mRNA levels were significantly higher in *Rab27a* siRNA-treated OVX mice (Supplementary Figure S9d and e). To further assess the effect of exosomes on miR-214 transfer from osteoclasts *in vivo*, histology images for Alp staining was performed and showed that the Alp level in bone surface of OVX siRNA mice was much higher compared with that in OVX NC mice (Figure 7k). A dynamic bone histomorphometric analysis of distal femurs showed that bone formation rate was increased in *Rab27a* siRNA-treated OVX mice (Figure 7l). Micro-computed tomography revealed that the ratio of bone volume to tissue volume, trabecular number and bone mineral density were upregulated and structure model index were downregulated after *Rab27a* siRNA treatment (Figure 7m). Under basal conditions, inhibition of exosome release by *Rab27a* siRNA treatment resulted in reduced circulation of miR-214 levels both in the serum and exosomes (Supplementary Figure S9f). *Alp*, *Bglap* and *Col1α1* mRNA levels were increased in bone and Alp^+^ cells (Supplementary Figure S9g and h). However, *Nfatc1*, *Acp5* and *Ctsk* mRNA levels were also upregulated in whole femurs and Oscar^+^ cells (Supplementary Figure S9i and j). The results of micro-computed tomography also showed that bone volume to tissue volume and bone mineral density were increased and structure model index was reduced in the *Rab27a* siRNA-treated control mice (Supplementary Figure S9k). These data indicate that inhibition of exosome secretion via downregulation of *Rab27a* levels enhances the osteoblast activity and increases bone mass.

## Discussion

**Figure 8:**
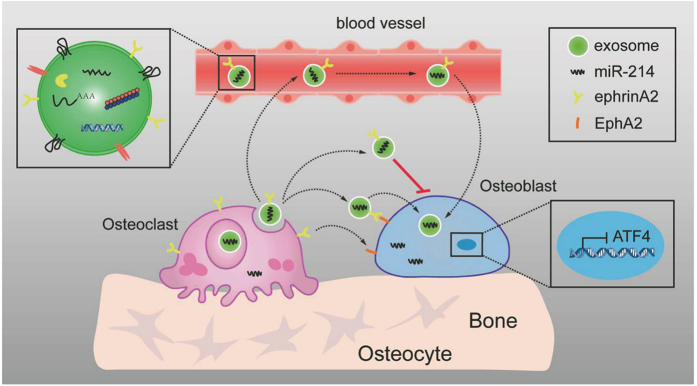
Model by which miR-214-containing exosomes from osteoclasts mediate crosstalk between osteoclasts and osteoblasts. In the course of osteoclastogenesis, miR-214 can be secreted in the form of exosomes, which could be transferred into osteoblasts via ephrinA2/EphA2 recognition to inhibit osteoblasts function. miR-214-containing exosomes can also be secreted into blood, and circulating miR-214 may be used as a biomarker for bone loss.

Bone remodeling is a fine-tuned process requiring the physiological coupling of osteoclasts and osteoblasts. In this study, we identified a mechanism of osteoclast-mediated suppression of osteoblasts through miRNA-containing exosomes. Osteoclasts secrete miR-214-enriched exosomes. These exosomes can be incorporated into osteoblasts via ephrinA2/EphA2 molecules. Consequently, miR-214 is transferred into osteoblasts to inhibit their activity. miR-214 levels in circulating exosomes were significantly upregulated in osteoclast-specific miR-214 transgenic mice. Simultaneously, miR-214 levels in osteoblasts were also increased and osteoblast function inhibited. *In vitro*, these exosomes exhibit an inhibitory effect on osteoblast function. Exosomes from both osteoporotic patients and OVX mice contained higher levels of miR-214 and ephrinA2 protein, suggesting that exosomes exert a marked inhibitory function on osteoblast activity. In OVX mice, prevention of exosome formation by downregulation of *Rab27a* increased osteoblast activity. In summary, we describe a new mechanism for the crosstalk between osteoblasts and osteoclasts, locally or at a distance, through exosome-mediated miRNA transfer from osteoclasts to osteoblasts (Figure 8).

It has been demonstrated that osteocytes and osteoblasts are major sources of both RANKL and osteoprotegerin and are therefore not only essential for the maintenance and formation of bone matrix, but simultaneously orchestrate the processes of osteoclast differentiation and bone resorption [6, 7]. However, little is known about the regulatory roles of osteoclasts in these processes. In this study, we identified a novel mode of osteoclast–osteoblast communication, in which miR-214 is secreted by osteoclasts encapsulated in exosomes and then transferred to osteoblasts to inhibit their function. Osteoclasts highly express miR-214, the expression of which is further increased during RANKL-induced osteoclastogenesis. Therefore, one can speculate that, given the function of osteoblast upregulation, more RANKL is available to stimulate osteoclastogenesis, which, in turn, produces more miR-214 to reduce osteoblast differentiation, thus forming a negative feedback loop to maintain bone formation and resorption homeostasis.

Exosomes are membrane vesicles 30–100 nm in diameter that are released into the extracellular environment. Exosomes can horizontally transfer functional proteins, mRNAs and miRNAs to neighboring cells and serve as mediators of intercellular communication [58]. However, the factor that directs the specificity of the exosomes in this process remains unclear. It has been reported that exosomes bind to cells through receptor–ligand interactions [18]. Here, we identified a pair of key proteins involved in this pathway. Osteoclast-derived exosomes bind to osteoblasts through an ephrinA2/EphA2 interaction to suppress osteoblast function by releasing miR-214. The osteoclast and osteoblast transwell coculture experiments demonstrated that miR-214-containing exosomes serve as mediators of osteoclast–osteoblast crosstalk, and this process is independent of direct cell contact. Eph receptors are members of the receptor tyrosine kinase family, and their cell surface ligands (ephrins) are involved in a variety of cell communication processes [59]. The ephrinA2-EphA2 interaction inhibits osteoblast differentiation during the initiation phase of bone remodeling, but stimulates osteoclast formation [57]. Inhibition of osteoblast differentiation by ephrinA2-EphA2 could be mediated by increased RhoA activity. Our results demonstrate that miRNAs are also involved in this process via exosome transportation. The encapsulation and secretion of exosomes from osteoclasts could represent an excellent target for therapeutic approaches to keep bone homeostasis.

Circulating miR-214 levels in exosomes and serum were increased significantly in osteoclast-specific transgenic mice, suggesting that osteoclast-secreted exosomes exist not only in the bone microenvironment but can also enter the blood. In osteoporotic patients, the levels of circulating miR-214 were much higher compared with those in non-osteoporotic individuals. Moreover, miR-214 was upregulated in both exosomes and serum in blood from hindlimb suspension and OVX-induced osteoporotic mice. These exosomes exerted an inhibitory function on osteoblast activity. Taken together, these results indicate that miRNA-mediated communication between osteoblasts and osteoclasts occurs in the bone microenvironment, yet at a distance, by trafficking of exosomes through the systemic circulation in a similar manner as hormones. miR-214 levels in the blood could serve as a biomarker for osteoporosis diagnostic purposes.

We attempted to decrease the level of circulating exosomes in OVX mice by inhibiting their release through *Rab27a* RNA interference. Upregulation of osteoblast activity was observed *in vivo* [60]. Unfortunately, osteoclast activity was also enhanced, and miR-214 levels were increased in osteoclasts following a reduction in *Rab27a* levels. It has been reported that the expression of *Rab27a* is upregulated during osteoclast differentiation. *Rab27a* deficiency induces multinucleated and giant cells, in which mRNA levels of various osteoclast marker genes, such as *RANK*, *Ctsk* and *CTR*, are significantly higher compared with WT osteoclasts. However, bone resorption activity in *Rab27a*-deficient osteoclasts is impaired owing to abnormal actin ring formation and altered localization of LAMP2 and Ctsk [61]. We also found similar changes in osteoclasts upon *Rab27a* knockdown. These results suggest that circulating exosomes have an important role in the regulation of osteoblast activity in the OVX model. Further experiments are required to develop a specific delivery system to inhibit exosome release specific to osteoclasts.

At present, several miRNAs in the serum are reportedly related to osteoporosis [62–64]. However, little is known about the relationship between these miRNAs and osteoporosis, and their biological origins or functions. In this study, we not only identified a correlation between miR-214 levels in serum and osteoporosis but also clarified the mechanism of its regulation on the balance between osteoblasts and osteoclasts. Understanding the role and action of miRNAs from exosomes is of utmost importance in the field of osteoporosis biomarker discovery and for the development of new biomedical applications for osteoporosis therapeutics.

## Materials and Methods

### Animals

All mice used in the experiments were bred and maintained at the SPF Animal Research Building of China Astronaut Research and Training Center (12-h light, 12-h dark cycles, temperature controlled for 23 °C and free access to food and water). Animals were fed with standard maintenance rodent diet (Beijing Keao Xieli Feed Co. Ltd, Beijing, China). The mice used in this study were C57BL/6 J background. Mice were killed by injection with Avertin (2.5% 2, 2, 2-tribromoethanol; Sigma-Aldrich, St Louis, MO, USA). The experimental procedures were approved by the Animal Care and Use Committee of China Astronaut Research and Training Center, and all animal studies were performed according to approved guidelines for the use and care of live animals. For this study, we used osteoblast-specific miR-214 transgenic mice (OB-TG214) and osteoclast-specific miR-214 transgenic mice (OC-TG214), which were reported previously [42, 55].

### Cell culture

The RAW 264.7 cell line was maintained in DMEM (Dulbecco's modified Eagle's medium; Invitrogen, Carlsbad, CA, USA) with 10% fetal bovine serum and 1% penicillin and streptomycin. The hFOB 1.19 cell line (transfected with SV40) was maintained in DMEM/F12 (Invitrogen) with 10% fetal bovine serum and 1% penicillin and streptomycin. The MC3T3-E1 clone 4 cell line was maintained in α-MEM (Invitrogen) containing 10% fetal bovine serum and 1% penicillin and streptomycin. The CD14^+^ PBMCs (peripheral blood mononuclear cells) was obtained under the published protocol [39]. The CD14^+^ PBMC was induced with 25 ng ml^−1^ recombinant human M-CSF and 30 ng ml^−1^ RANKL (R&D Systems, Minneapolis, MN, USA). These cells were maintained under standard cell culture conditions of 5% CO_2_ and 95% humidity. The culture medium was replaced with fresh medium every 2 days.

### Apoptotic body, microvesicle and exosome isolation

AB, MV and exosome were collected by differential centrifugations. Exosomes were collected by differential centrifugations as previously described with some modifications [65]. Briefly, RANKL-induced RAW 264.7 cells were cultured in exosome-free medium. Culture medium was supplemented with fetal bovine serum previously depleted of exosomes by ultracentrifugation at 120 000 *g* for 90 min. Then, cell culture supernatant containing exosomes were harvested by centrifugation at 300 *g* for 10 min at 4 °C to eliminate cells, and then at  2000 *g* for 20 min to obtain apoptotic body and at 20 000 *g* for 30 min to obtain microvesicles. At each step, the supernatant was transferred to new tubes and the pellets immediately resuspended in either phosphate-buffered saline (PBS) or rinsed with PBS. The resulting supernatant was filtered through a 0.22-μm membrane to remove particles larger than 200 nm. Exosomes were pelleted via ultracentrifugation at 120 000 *g* for 70 min at 4 °C, washed in 50 ml PBS and pelleted again by ultracentrifugation, and the pellets resuspended in PBS. Exosomes were used immediately or stored at −80 °C.

For RNA and protein, the exosome was isolated with Total Exosome Isolation Kit (Invitrogen). Exosome from the culture medium was incubated with the indicated reagents for overnight at 2–8 °C, the mixture was then centrifuged at 10 000 *g* for 1 h at 2–8 °C. Exosome from the serum was incubated with the indicated reagents for 30 min at 2–8 °C, the mixture was then centrifuged at 10 000 *g* for 10 min at room temperature. RNA from exosomes was extracted by Trizol (Invitrogen). TSG101 and HSP70 were detected by western blot as exosome markers.

### FACS analysis of exosomes

For fluorescence-activated cell sorter (FACS) analysis, exosomes were coated onto 4-μm-diameter aldehyde/sulfate latex beads, using a previously described method [66]. Briefly, 5 μg exosomes was incubated with 10 μl 4 -μm-diameter aldehyde/sulfate latex beads for 15 min at room temperature in PBS. The mixture was then transferred to 1 ml PBS with gentle shaking for 2 h. After centrifugation, the pellet was blocked by incubation with 110 μl of 1 m glycine (100 mm final) for 30 min. Exosome-coated beads were washed two times in PBS and resuspended in 100 μl PBS. Afterwards, beads were incubated with PE-labeled CD63 antibody for 1 h at room temperature in the dark. Beads were analyzed by flow cytometry using a FACSCalibur flow cytometer (Becton Dickinson, Mountain View, CA, USA) and the Flow Jo software (Tree Star, Ashland, OR, USA).

### Dynamic light scattering

Dynamic light scattering (DLS) measurements were performed using a Malvern Zetasizer Nano ZS (Malvern, Worcestershire, UK). Cell conditioned medium samples were allowed to equilibrate at 25 °C for 5 min to ensure temperature homogeneity before making five measurements, each consisting of five individual runs. The viscosity and refractive index values used were 0.8872 cP and 1.330, respectively, as provided in the Zetasizer software (v.2.0). The refractive index and absorption values for the exosomes used to calculate volume and number size distributions were 0.2 and 0.01, respectively, as provided in the Zetasizer software. Exosome sizing by DLS was performed using the intensity versus size mode.

### miRNA microarray analysis

RANKL-induced RAW 264.7 cells and RAW 264.7 cell-derived exosomes, purified by differential centrifugations, were disrupted with Trizol. RNA isolation was performed with the miRNeasy Mini Kit (Qiagen, Hilden, Germany). The miRNA was amplified and hybridized to Illumina expression profiling microarrays according to the manufacturer’s directions. Briefly, RNA was poly-A tailed and reverse transcribed from a polydT-tailed primer to generate biotinylated cDNA. The cDNA underwent allele-specific extension with miRNA-specific oligos for all targeted sequences, followed by amplification with a cyanine 3 fluorophore-labeled primers. The double-stranded PCR product was bound to magnetic beads and denatured with 0.1 n NaOH. The fluorophore-labeled strand was hybridized to the miRNA microarray containing 1536 IllumiCode sequences attached to 3 μm beads. Hybridization was performed overnight with a 60-45 °C temperature ramp, washed to remove excess label and dried in a vacuum dessicator before imaging by laser excitation of the fluorochrome-labeled sample. Fluorescence intensity was quantitatively imaged with the iScan system (Illumina, San Diego, CA, USA) for expression analysis.

### Electron microscopy

Purified exosomes from RANKL-induced RAW 264.7 cells were resuspended in PBS and fixed with 2% paraformaldehyde for 30 min at room temperature. Eight microliters of mixture were then dropped onto EM grids that had been pretreated with UV light to reduce static electricity. After drying for 30 min, exosomes were stained two times (6 min each) with 1% uranyl acetate. The dried grids were examined using an HT7700 (Hitachi, Tokyo, Japan) transmission electron microscope at 120 kV.

### Exosome labeling

The fluorescent dye 3, 3′-dioctadecyloxacarbocyanine perchlorate was purchased from Invitrogen and was used to label exosomes and cell membrane, respectively. Purified exosomes were incubated with fluorescent dye 3′-dioctadecyloxacarbocyanine perchlorate (5 mm) for 15 min at 37 °C, and then ultracentrifuged at 120 000 *g* for 90 min to remove free dye. After being washed two times in PBS with 120 000 *g* centrifugation, the labeled exosomes were resuspended in PBS before being used.

### MiRNA extraction

Total RNA from the collected cells was extracted using the miRNeasy Mini Kit (Qiagen). Briefly, the cells were collected in a reaction tube, lysed with QIAzol and mixed with chloroform. After being centrifuged at 12 000 *g* for 15 min at 4 °C, the upper aqueous phase was transferred to an RNeasy Mini spin column in a 2 ml collection tube and mixed with 100% ethanol. After being washed with 700 μl Buffer RWT and 500 μl Buffer RPE, the total RNA was collected for real-time PCR analysis.

### RT-PCR and qRT-PCR

Total RNA from bone tissues or cells was extracted with TRIzol reagent (Invitrogen) as per the manufacturer's instructions. The cell samples were washed with PBS before being treated with TRIzol. RNA (0.5 μg) was reverse transcribed with PrimeScript RT reagent Kit (TaKaRa, Dalian, China) according to the manufacturer's instructions. Stem-loop RT-PCR was used for the quantification of miR-214. Two microliters of cDNA were used for detecting mRNA and miRNA expression by qPCR using SYBR Premix Ex Taq II Kit (TaKaRa). *Gapdh* was used as a normalization control for mRNA and *RNU6* was used as a normalization control in all miRNA measurements.

### Quantification of miR-214 and miR-214* copies

First, the standard curve was made according to the absolute copies of miR-214 or miR-214* and its Ct value, respectively. miR-214 was diluted into 0.2, 2, 20, 200, 2 000 and 20 000 nm, and 1 μl was reverse transcribed into 10 μl cDNA. miR-214* was diluted into 0.23, 2.3, 23, 230, 2 300 and 23 000 nm, and 1 μl was reverse transcribed into 10 μl cDNA. Two microliters of diluted cDNA (1:5) were used for miR-214 absolute quantitative analysis by qRT-PCR. Using the standard curves, we quantitated miR-214 and miR-214 star copies in osteoblast, osteoclast and exosomes from miR-214 star-transfected osteoclasts.

*Mouse primers*: *Gapdh*, forward: 5′-AACATCAAATGGGGTGAGGCC-3′ and reverse: 5′-GTTGTCATGGATGACCTTGGC-3′; *Nfatc1*, forward: 5′-ACGCTACAGCTGTTCATTGG-3′ and reverse: 5′-CTTTGGTGTTGGACAGGATG-3′; *Acp5*, forward: 5′-GCGACCATTGTTAGCCACATACG-3′ and reverse: 5′-CGTTGATGTCGCACAGAGGGAT-3′; *Clcn7*, forward: 5′-GTCCTTCAGCCTCAGTCG-3′ and reverse: 5′-ACACAGCGTCTAATCACAAC-3′; *Mmp9*, forward: 5′-GCGGCCCTCAAAGATGAACGG-3′ and reverse: 5′-GCTGACTACGATAAGGACGGCA-3′; *Ctsk*, forward: 5′-GCGTTGTTCTTATTCCGAGC-3′ and reverse: 5′-TTTCCCGTTCACCGTCCAC-3′; *Alp*, forward: 5′-ATCTTTGGTCTGGCTCCCATG-3′ and reverse: 5′-TTTCCCGTTCACCGTCCAC-3′; *Bglap*, forward: 5′-CCAAGCAGGAGGGCAATA-3′ and reverse: 5′-TCGTCACAAGCAGGGTCA-3′; *Col1α1*, forward: 5′-GGGACCAGGAGGACCAGGAAGT-3′ and reverse: 5′-GGAGGGCGAGTGCTGTGCTTT-3′; *Efna2*, forward: 5′-AGCATGCAAATGAGGCCTGGTGATG-3′ and reverse: 5′-GAGTCTGAGGGTGAGGGCTTCC-3′; *Epha2*, forward: 5′-ACTGAAAGCGGGCTACACTGAGAA-3′ and reverse: 5′-AGGCGGATGATATTGTGGTGGCTA-3′; *Sema4d*, forward: 5′-CCTGGTGGAGTGTTGAGAAC-3′ and reverse: 5′-GCA AGGCCGAGTTAA AGAT-3′; mmu-miR-155-5p, forward: 5′-TTAATGCTAATTGTGATAGGGGTGC-3′ and mmu-miR-148a-3p, forward: 5′-TCAGTGCACTACAGAACTTTGTG-3′; mmu-miR-320-3p, forward: 5′-AAAAGCTGGGTTGAGAGGGC-3′; mmu-miR-92a-3p, forward: 5′-TATTGCACTTGTCCCGGCCT-3′; mmu-miR-199a-3p, forward: 5′-ACAGTAGTCTGCACATTGGTTAG-3′; mmu-miR-23a-3p, forward: 5′-ATCACATTGCCAGGGATTTCCG-3′; mmu-miR-21a-5p, forward: 5′-TAGCTTATCAGACTGATGTTGAGC-3′; mmu-miR-125a-5p, forward: 5′-TCCCTGAGACCCTTTAACCTGT-3′; mmu-miR-378a-3p, forward: 5′-ACTGGACTTGGAGTCAGAAGG-3′; mmu-miR-27a-3p, forward: 5′-TTCACAGTGGCTAAGTTCCGC-3′; mmu-miR-16-5p, forward: 5′-TAGCAGCACGTAAATATTGGCG-3′; mmu-miR-183-5p, forward: 5′-TATGGCACTGGTAGAATTCACTG-3′; mmu-miR-214-3p, forward: 5′-ACAGCAGGCACAGACAGGC-3′; miRNA universal reverse, 5′-GAATCGAGCACCAGTTACGC-3′.

*Human primers*: *ALP*, forward: 5′-GTGAACCGCAACTGGTACTC-3′ and reverse: 5′-GAGCTGCGTAGCGATGTCC-3′; *BGLAP*, forward: 5′-GGCGCTACCTGTATCAATGG-3′ and reverse: 5′-GTGGTCAGCCAACTCGTCA-3′; COL1a1, forward: 5′-GAGGGCCAAGACGAAGACATC-3′ and reverse: 5′-CAGATCACGTCATCGCACAAC-3′; GAPDH, forward: 5′-ACAACTTTGGTATCGTGGAAGG-3′ and reverse: 5′-GCCATCACGCCACAGTTTC-3′.

### Circulating miRNA extraction and detection by qRT-PCR

MiRNA from 200 μl serum was extracted using the miRNeasy Serum/Plasma Kit (Qiagen) according to the manufacturer’s recommendations. Brifely, we supplemented the samples (after the addition of QIAzol) with 3.5 μl miRNeasy Serum/Plasma Spike-In Control (1.6×10^8^ copies per μl working solution) (Qiagen). We demonstrate that the cel-miR-39 can be used for normalization of the RNA preparation. The amount and purity of RNA was estimated by Quawell Micro Volume spectrophotometer (Quawell, San Jose, CA, USA). Subsequently, miRNA was transcribed to cDNA using the miScript II RT Kit (Qiagen). Diluted cDNA (1:10) were used for detecting miRNA expression by qPCR using the miScript SYBR Green PCR Kit with miR-214 miScript Primer Assay (Qiagen). The relative expression level of miR-214 was determined by the cycle number via qPCR, with their levels normalized to the average of cel-miR-39 using the 2^−ΔΔCT^ method.

### Western blot analysis

Cells were lysed in lysis buffer (50 mm Tris, pH 7.5, 250 mm NaCl, 0.1% sodium dodecyl sulfate, 2 mm dithiothreitol, 0.5% NP-40, 1 mm phenylmethylsulfonyl fluoride and protease inhibitor cocktail) on ice for 30 min. Protein fractions were collected by centrifugation at 15 000 *g* at 4 °C for 30 min and then subjected to sodium dodecyl sulfate-polyacrylamide gel electrophoresis and transferred to polyvinylidene difluoride (PVDF) membranes. The membranes were blocked with 5% bovine serum albumin and incubated with specific antibodies overnight. A horseradish peroxidase-labeled secondary antibody was added and visualized using an Enhanced Chemiluminescence Kit (Thermo Pierce, Waltham, MA, USA). The antibodies used are as follows: TFIIB (Cell Signaling Technology, Danvers, MA, USA), LaminA/C (CST), β-actin (CST), CD63 (Bioss, Beijing, China), tartrate-resistant acid phosphatase (Bioss), *Rab27a* (Bioss), ATF4 (Abcam, Cambridge, UK), ephrinA2 (R&D System), EphA2 (Santa Cruz Biotechnology, Santa Cruz, CA, USA), HSP70 (Santa Cruz Biotechnology), TSG101(C-2, Santa Cruz Biotechnology), RhoA (CST) and GAPDH (Santa Cruz Biotechnology) to examine the concentrations of proteins in the lysates, respectively. The ratios of the protein band intensities relative to that of GAPDH were calculated for each sample using Image J (Bethesda, MD, USA). Active RhoA were detected by Configuration-Specific Monoclonal Antibody-Based RhoA Activity Assay Kit (NewEast, Malvern, PA, USA) according to the manufacturer’s protocol.

### Alkaline phosphatase staining

Alkaline phosphatase staining was monitored using a Vector Blue Substrate Kit (procedure number SK-5300; Vector Laboratories, Burlingame, CA, USA). According to the protocol, MC3T3-E1 or hFOB1.19 cells were incubated with the substrate working solution for 20–30 min. The whole procedure was protected from light. After 2 min of rinsing in deionized water, slides were treated with Mayer’s hematoxylin solution for 10 min.

### Cell sorting with flow cytometry

The bone marrow cells and bone marrow stromal cells were collected from the femur and tibia of WT and TG214 mice. Antibody to mouse Alp (R&D Systems) and antibody to mouse Oscar (Santa Cruz Biotechnology) antibodies were used for FACS according to the following protocol. After washing with PBS and 1% bovine serum albumin, the cells were directly stained with antibody to Alp (1:50) and then stained with goat anti-mouse IgG-FITC (1:100; R&D Systems) or were incubated with antibody to Oscar (1:40) and then stained with donkey anti-goat IgG-PE (1:100; R&D Systems). After that, stained cell populations were used for FACS. The obtained selected Alp^+^ and Oscar^+^ cell populations were used for total RNA extraction and qRT-PCR analysis.

### Assessment of new bone formation

The mice were injected intraperitoneally with calcein green (10 mg kg^−1^ body weight) in a time sequence of 10 and 2 days before being killed. The lumbar vertebal 5 bone was harvested for undecalcified histology analysis. Unstained 3-μm sections are examined using fluorescence microscopy.

### OVX-induced osteoporotic mouse model

All the female C57BL/6 J mice used were maintained under standard animal housing conditions (12-h light, 12-h dark cycles and free access to food and water). The mice were OVX or sham-operated at 3 months of age. At 8 months after surgery (11 months of age), serum and bone tissues from sham-operated and OVX mice were collected.

### Hindlimb suspension mouse model

The hindlimb-unloading procedure was achieved by tail suspension. Briefly, the 3-month-old male C57BL/6 J mice were individually caged and suspended by the tail using a strip of adhesive surgical tape attached to a chain hanging from a pulley. The mice were suspended at a 30° angle to the floor, with only the forelimbs touching the floor; this allowed the mice to move and access food and water freely. The mice were subjected to hindlimb unloading through tail suspension for 28 days. After killing, the serum and whole-bone tissues were collected.

### Rescue treatment by *Rab27a* siRNA in OVX mice

Female C57BL/6 J mice were OVX or sham operated at 3 months of age and left untreated until they were 11 months old. OVX and sham mice were treated with *Rab27a* siRNA and NC (5 μg per mouse) by tail vein injection two times per week. The siRNA was packed into amphiphilic methoxy poly(ethylene glycol)-poly(lactic-co-glycolic acid) copolymer nanoparticles before injection. The nanoparticles was obtained from the National Center for Nanoscience and Technology of China (Beijing, China), as reported [67, 68].

### Immunohistochemistry

The fifth lumbar vertebra of mice were fixed with 4% buffered formalin and embedded with paraffin after decalcification with 10% EDTA and 5–7 μm sections were prepared on a rotation microtome. Paraffin-embedded sections were deparaffinized in xylene, and rehydrated. The samples were stained with hematoxylin–eosin or ALP antibody (R&D System) overnight. The samples were also counterstained with 0.1% 30% hematoxylin. The sections were then dehydrated and mounted in Permount.

### Micro-computed tomography analysis

High-resolution micro-CT analyses were performed on the distal femurs using a model of μ40 scanco (SCANCO Medical, Bruttisellen, Switzerland). In the femurs, the trabecular bone proximal to the distal growth plate was selected for analyses within a conforming volume of interest (cortical bone excluded) commencing at a distance of 315 μm from the growth plate and extending a further longitudinal distance of 1260 μm in the proximal direction.

### Human serum sample collection

The serum of 42 osteoporotic and 23 non-osteoporotic patients were collected from clinical setting (309 Hospital of PLA, Beijing, China). The men were recruited at between 50 and 90 years of age, and the women were recruited over 5 years postmenopause. Subjects with malignancy, diabetes or other severe diseases in the previous 5 years were excluded from our study (exclusive criteria). The classification of the patients in the osteoporotic and the non-osteoporotic group was based on dual-energy X-ray absorptiometry evaluation. We measured the *T*-score for bone mineral density at hip joint of men and spine of women. A *T*-score of −2.5 or lower qualifies as osteoporosis. Others are non-osteoporotic patients. All the clinical procedures were approved by the Committees of Clinical Ethics in the Institute of Orthopedics, 309 Hospital of Chinese People’s Liberation Army (Beijing, China).

### Statistical analysis

All numerical data are expressed as the mean±s.e.m. Statistical differences among groups were analyzed by one-way analysis of variance with a *post hoc* test to determine group differences in the study parameters. All statistical analyses were performed with SPSS software (version 13.0 for windows, Armonk, NY, USA) and Prism software (GraphPad prism for windows, version 5.01, Nashville,TN, USA). Statistical differences between two groups were determined by the Student’s *T*-test. *P*<0.05 was considered statistically significant.
