# Supplementary material for: Osteoclast-derived microRNA-containing exosomes selectively inhibit osteoblast activity
Source: Cell Discov. 2016 May 31;2:16015–. doi: 10.1038/celldisc.2016.15 (PMC4886818; doi:10.1038/celldisc.2016.15)
Supplement: Supplementary Figure S1 [file celldisc201615-s1.pdf]

Supplementary Figure 1

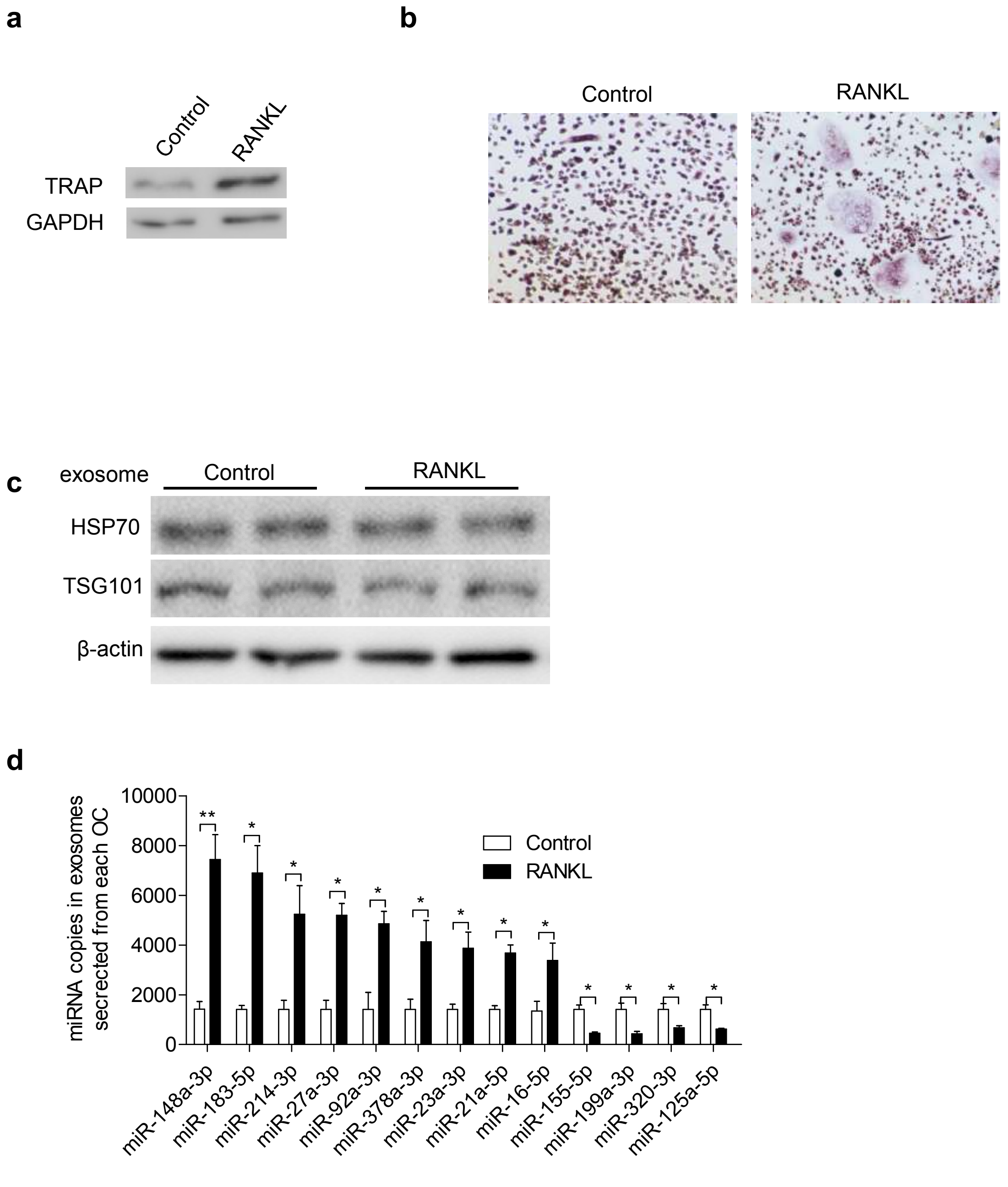

Supplementary Figure 1. Tartrate-resistant acid phosphatase (TRAP) protein levels during the process of osteoclastogenesis and miR-214 levels in exosomes.

(**a**) TRAP protein levels in RAW 264.7 cells with and without RANKL (50 ng/ml) induction for 2 days were analyzed by western blot. (**b**) Representative images of TRAP staining of RAW 264.7 cells with and without RANKL treatment. (**c**) The protein levels of HSP70 , TSG101 and β-actin in exosomes secreted by RANKL-induced RAW 264.7 cells for 2 days were analyzed by western blot. (**d**) miRNAs copies in exosomes secreted from each osteoclast cell were analyzed by qRT-PCR and calculated according to the standard curves. The data represent the mean ± SEM of three independent experiments. **P*<0.05, ***P*<0.01.
