# Supplementary material for: Osteoclast-derived microRNA-containing exosomes selectively inhibit osteoblast activity
Source: Cell Discov. 2016 May 31;2:16015–. doi: 10.1038/celldisc.2016.15 (PMC4886818; doi:10.1038/celldisc.2016.15)
Supplement: Supplementary Figure S2 [file celldisc201615-s2.pdf]

Supplementary Figure 2

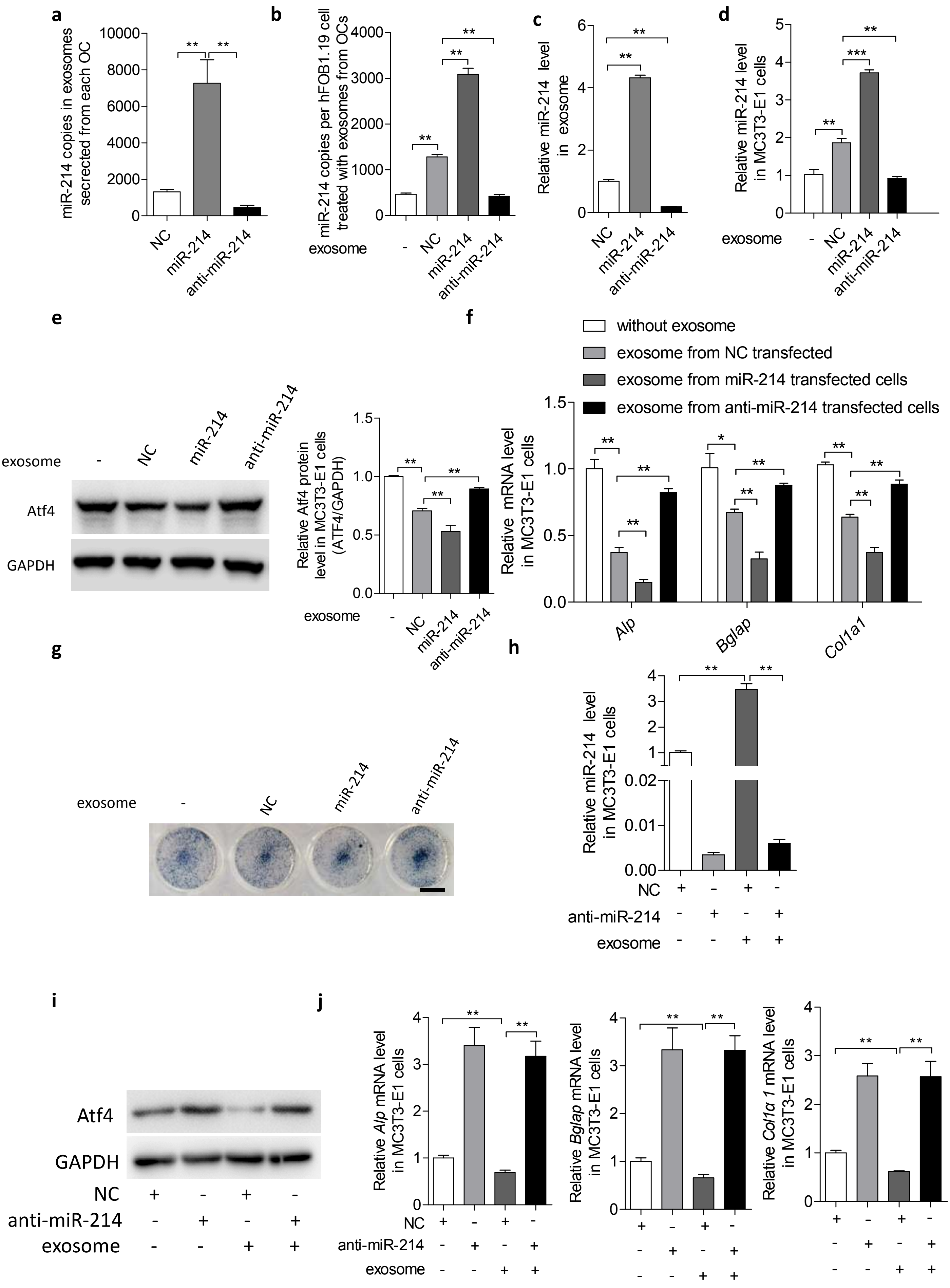

Supplementary Figure 2

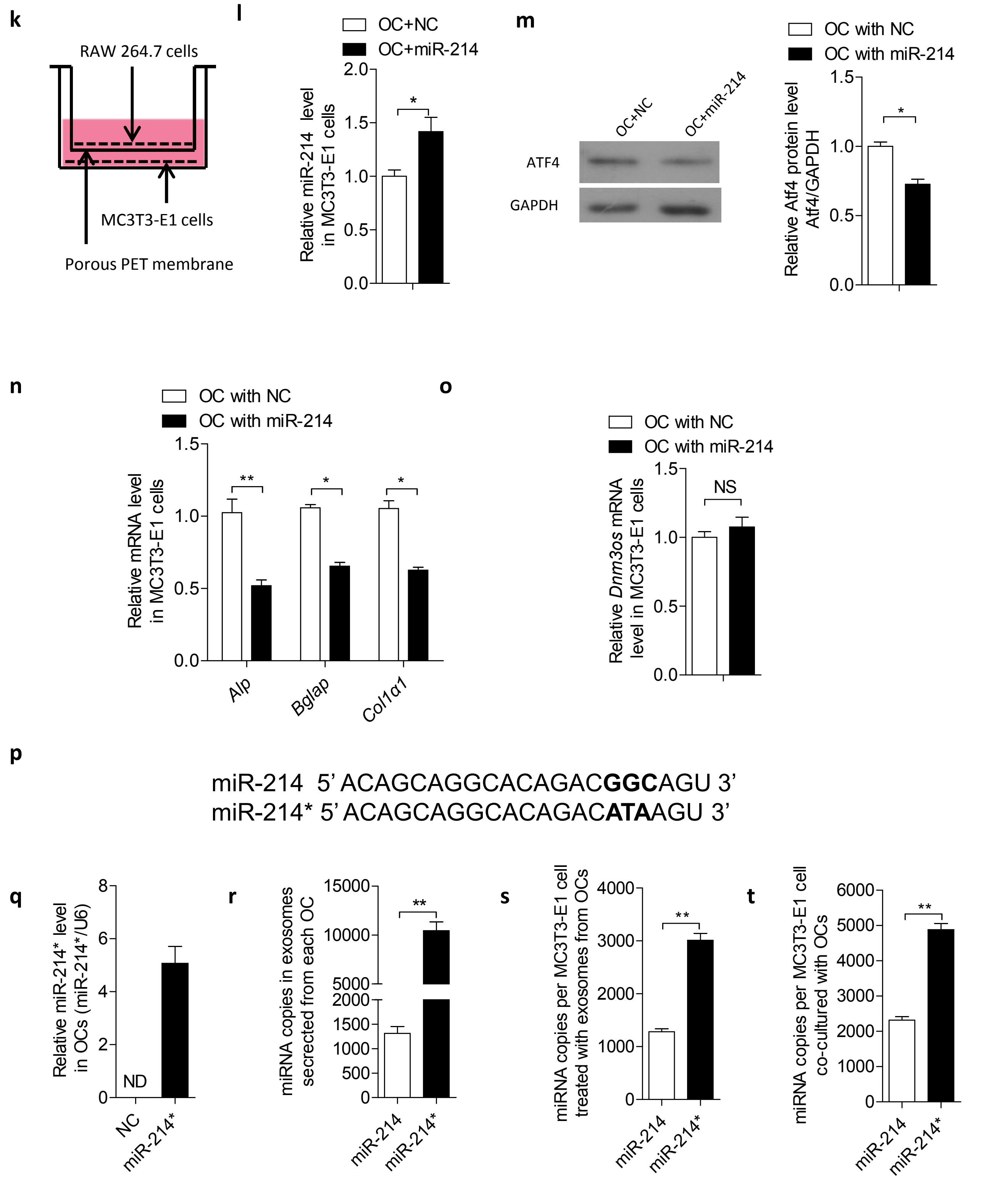

Supplementary Figure 2. miR-214 transfer from osteoclasts to osteoblasts to inhibit osteoblast function.

(**a**) miR-214 copies in exosomes secreted from each osteoclast cell were analyzed by qRT-PCR and calculated according to the standard curves. (**b**) miR-214 copies in each hFOB1.19 cell were analyzed by qRT-PCR and calculated according to the standard curves. hFOB1.19 cells (10^5) were incubated with exosomes secreted from osteoclasts (10^6) transfected with 300 pmol NC, miR-214 or anti-miR-214. (**c**) QRT-PCR analysis of miR-214 levels in exosomes from the medium of RAW 264.7 cells. These cells were transfected with NC (negative control), miR-214 (miR-214 mimics) or anti-miR-214 (the inhibitor of miR-214) for 2 days under the treatment with RANKL. MiR-214 levels were normalized to *RNU6*. (**d**) miR-214 level in MC3T3-E1 cells was analyzed by qRT-PCR after incubation for 2 days with or without exosomes secreted (5 µg/ml) from RAW 264.7 cells transfected with NC, miR-214 or anti-miR-214 for 2 days under the treatment with RANKL. (**e**) Atf4 protein levels in MC3T3-E1 cells were analyzed by western blot after incubation with or without exosomes and were normalized to GAPDH. (**f**) *Alp*, *Bglap* and *Col1α1* mRNA levels in MC3T3-E1 cells were analyzed by qRT-PCR after incubation with or without exosomes. The transcripts levels were normalized to *Gapdh*. (**g**) Representative images of Alp staining of osteoblasts after incubation with or without exosomes for 6 days. (**h**) miR-214 level in MC3T3-E1 cells was analyzed by qRT-PCR after transfection with anti-miR-214 or NC, and incubation with or without exosomes (5 µg/ml) secreted from osteoclasts. (**i**) Atf4 protein levels in MC3T3-E1 cells were analyzed by western blot in those groups and were normalized to GAPDH. (**j**) *Alp*, *Bglap* and *Col1α1* mRNA levels in MC3T3-E1 cells were analyzed by qRT-PCR in those groups. The transcripts levels were normalized to *Gapdh.*

(**k**) *In vitro* co-culture system was used where RANKL - induced RAW 264.7 cells were seeded in the top compartment, which was separated by a 0.4 μm porous membrane from MC3T3-E1 cells that were cultured in the bottom compartment. (**l**) The change of miR-214 levels in MC3T3-E1 cells after co-cultured with RANKL (50 ng/ml)-induced RAW 264.7 cells transfected with miR-214 mimics or NC for 2 days. (**m**) The change of Atf4 protein level in MC3T3-E1 cells after co-cultured with RANKL-induced RAW 264.7 cells transfected with miR-214 mimics or NC. (**n**) The changes of *Alp*, *Bglap*, *Col1α1* mRNA levels in MC3T3-E1 cells after co-cultured with OC transfected with miR-214 mimics or NC. Their levels were analyzed by qRT-PCR and normalized to *Gapdh*. (**o**) The change of *Dnm3os* mRNA level in MC3T3-E1 cells after co-cultured with OC transfected with miR-214 mimics or NC. *Dnm3os* mRNA level was analyzed by qRT-PCR and normalized to *Gapdh*. (**p**) The sequences of miR-214 and miR-214*. (**q**) Osteoclasts were transfected with miR-214* or not, collected after 48 h, then the levels of miR-214* were analyzed by miR-214* specific probe. (**r**) miR-214 and miR-214* copies in exosomes secreted from each osteoclast were analyzed by qRT-PCR and calculated according to the standard curves. Osteoclasts (10^5) were transfected with 60 pmol miR-214*. (**s**) miR-214 and miR-214* copies in each MC3T3-E1 cell were analyzed by qRT-PCR and calculated according to the standard curves. MC3T3-E1 cells ($2*10^5$) were incubated with exosomes secreted from osteoclasts (10^5) transfected with 60 pmol miR-214*. (**t**) miR-214 and miR-214* copies in each osteoblasts were analyzed by qRT-PCR and calculated according to the standard curves. MC3T3-E1 cells ($2*10^5$) were co-cultured with osteoclasts (10^5) transfected with 60 pmol miR-214*. The data represent the mean ± SEM of three independent experiments. *P<0.05, **P<0.01.
