# Supplementary material for: Osteoclast-derived microRNA-containing exosomes selectively inhibit osteoblast activity
Source: Cell Discov. 2016 May 31;2:16015–. doi: 10.1038/celldisc.2016.15 (PMC4886818; doi:10.1038/celldisc.2016.15)
Supplement: Supplementary Figure S3 [file celldisc201615-s3.pdf]

Supplementary Figure 3

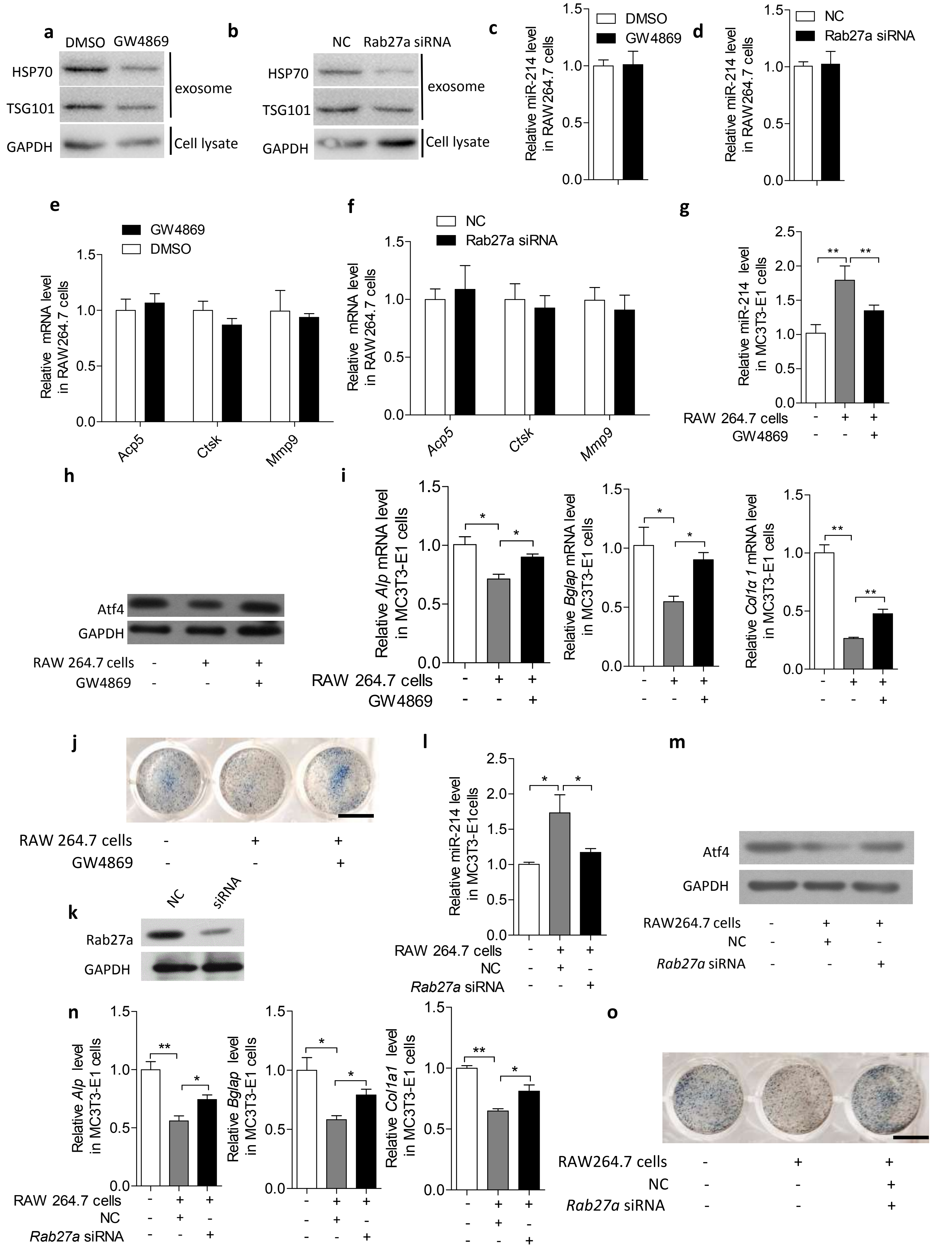

Supplementary Figure 3. Inhibition of exosome release from human osteoclast attenuates the inhibitory role of miR-214 on hFOB1.19 cell activity.

(**a,b**) The levels of HSP70 and TSG101 in exosomes secreted by RANKL-induced RAW264.7 cells treatment with DMSO, GW4869, Rab27a siRNA or NC for 2 days were analyzed by western blot. (**c,d**) miR-214 levels were analyzed by qRT-PCR in RAW 264.7 cells treatment with GW4869 DMSO, GW4869, Rab27a siRNA or NC. (**e,f**) *Acp5*, *Mmp9*, *Ctsk* mRNA levels were analyzed by qRT-PCR in RAW264.7 cells treatment with DMSO, GW4869, Rab27a siRNA or NC for 2 days. (**g**) miR-214 levels were analyzed by qRT-PCR in MC3T3-E1 cells which were co-cultured for 2 days with RANKL-induced RAW 264.7 cells after treatment with the nSMase inhibitor GW4869 (20 μM) .(**h**) Expression of Atf4 protein in MC3T3-E1 cells which were co-cultured with RANKL-induced RAW 264.7 cells for 2 days with or without GW4869 treatment (20 μM) for 2 days were analyzed by western blot and were normalized to GAPDH. (**i**) The mRNA levels of *Alp*, *Bglap* and *Col1α1* were analyzed by qRT-PCR in MC3T3-E1 cells which were co-cultured with RANKL-induced RAW 264.7 cells with or without GW4869 treatment. (**j**) Representative images of Alp staining of osteoblasts after co-cultured with RANKL-induced RAW 264.7 cells with or without GW4869 treatment. (**k**) Expression of Rab27a protein in RAW 264.7 cells transfected with Rab27a siRNA or NC were analyzed by western blot and normalized to GAPDH. (**l**) miR-214 level was analyzed by qRT-PCR in MC3T3-E1 cells which were co-cultured with RANKL-induced RAW 264.7 cells transfected with NC and Rab27a siRNA. (**m**) Expression of Atf4 protein in MC3T3-E1 cells which were co-cultured with RANKL-induced RAW 264.7 cells transfected with NC and Rab27a siRNA were analyzed by western blot and were normalized to GAPDH.

(**n**) *Alp*, *Bglap* and *Col1α1* mRNA level in MC3T3-E1 cells were analyzed by qRT-PCR in MC3T3-E1 cells which were co-cultured with RANKL-induced RAW 264.7 cells transfected with NC and Rab27a siRNA. (**o**) Representative images of Alp staining of osteoblasts after co-cultured for 6 days with RANKL-induced RAW 264.7 cells transfected with NC and Rab27a siRNA. The culture medium was replaced for fresh medium every 2 days. The data represent the mean ± SEM of three independent experiments. *P<0.05, **P<0.01.
