# Supplementary material for: Osteoclast-derived microRNA-containing exosomes selectively inhibit osteoblast activity
Source: Cell Discov. 2016 May 31;2:16015–. doi: 10.1038/celldisc.2016.15 (PMC4886818; doi:10.1038/celldisc.2016.15)
Supplement: Supplementary Figure S4 [file celldisc201615-s4.pdf]

Supplementary Figure 4

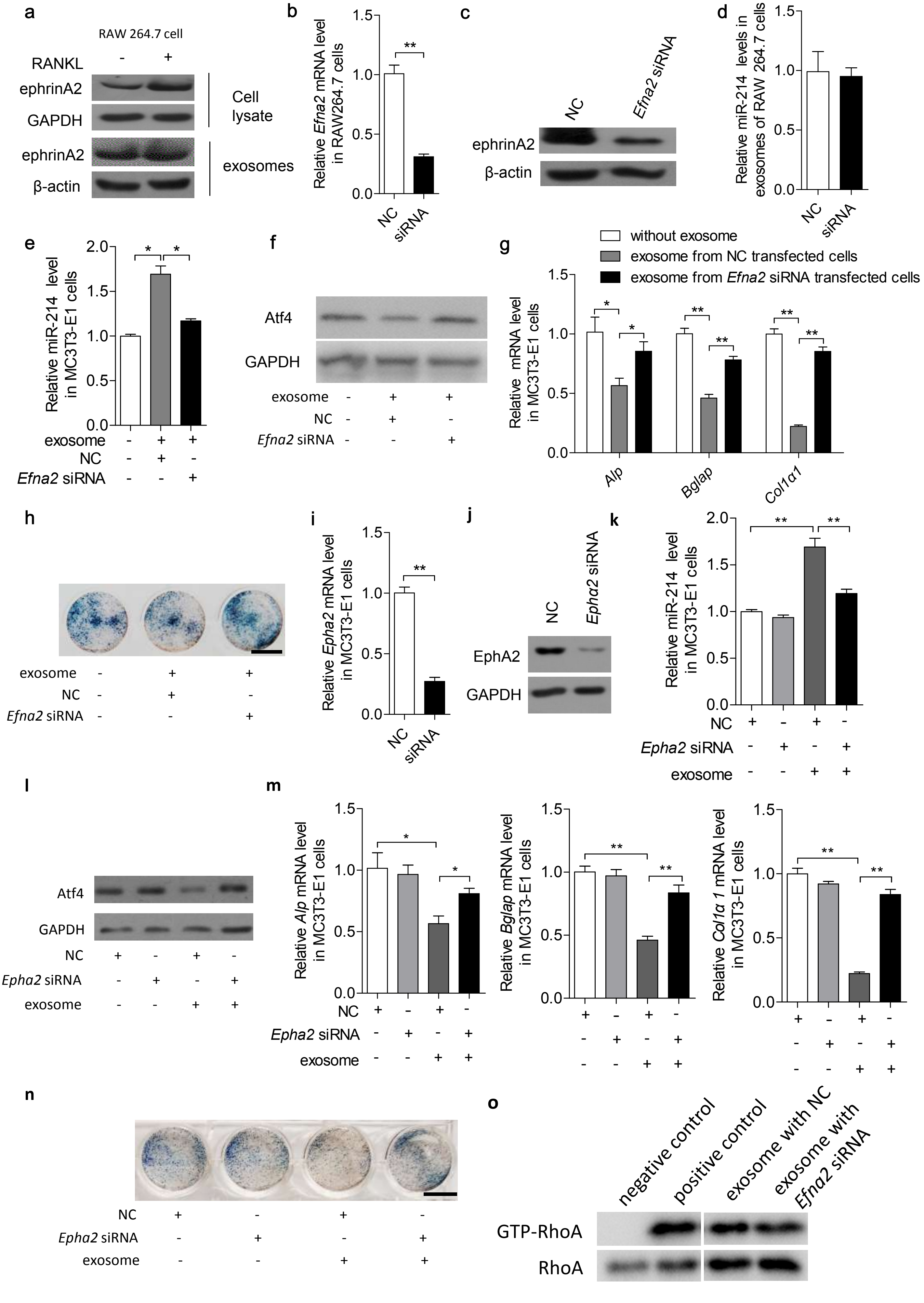

Supplementary Figure 4. Osteoclast-derived exosomes recognize osteoblasts through ephrinA2/EphA2.

(**a**) Immunoblotting analysis of ephrinA2 protein levels in cell lysate and exosomes from RAW 264.7 cells and RANKL-induced RAW 264.7 cells. EphrinA2 protein levels were normalized to GAPDH or β-actin. (**b**) *Efnα2* mRNA level in *Efnα2* siRNA-transfected RAW 264.7 cells were analyzed by qRT-PCR. The mRNA levels were normalized to *Gapdh*. (**c**) The change of ephrinA2 protein level in exosomes from *Efnα2* siRNA transfected RAW 264.7 cells was analyzed by western blot. EphrinA2 protein levels were normalized to β-actin. (**d**) The change of miR-214 levels in exosomes from *Efnα2* siRNA transfected RAW 264.7 cells with RANKL induction for 2 days were analyzed by qRT-PCR. (**e**) miR-214 levels in MC3T3-E1 cells were analyzed after incubation for 2 days without or with exosomes collected from the same number of *Efna2* siRNA transfected RAW 264.7 cells with RANKL induction for 2 days by qRT-PCR. (**f**) The change of Atf4 protein level in MC3T3-E1 cells after incubation without or with exosomes collected from the same number of *Efna2* siRNA transfected RAW 264.7 cells with RANKL induction for 2 days, Atf4 levels were analyzed by western blot and were normalized to GAPDH. (**g**) *Alp*, *Bglap* and *Col1α1* mRNA levels in MC3T3-E1 cells were analyzed after incubation without or with exosomes from *Efna2* siRNA transfected RAW 264.7 cells with RANKL induction for 2 days by qRT-PCR. (**h**) Representative images of Alp staining of osteoblasts after incubation for 6 days without or with exosomes from *Efnα2* siRNA transfected RAW 264.7 cells with RANKL induction for 2 days. The culture medium was replaced for fresh medium every 2 days. (**i**) *Ephα2* mRNA levels in MC3T3-E1 cells after transfected with *Epha2* siRNA were analyzed by qRT-PCR. (**j**) The change of EphA2 protein level in MC3T3-E1 cells was analyzed by western blot. EphA2 protein levels were normalized to GAPDH.

(**k**) miR-214 levels in MC3T3-E1 cells transfected with *Ephα2* siRNA were analyzed by qRT-PCR after incubation for 2 days with exosomes from RAW 264.7 cells with RANKL induction for 2 days. (**l**) The change of Atf4 protein level in *Epha2* siRNA-transfected MC3T3-E1 cells after incubation for 2 days with exosome from RANKL-induced RAW 264.7 cells, Atf4 levels were analyzed by western blot and were normalized to GAPDH. (**m**) *Alp*, *Bglap* and *Col1α1* mRNA levels in *Epha2* siRNA-transfected MC3T3-E1 cells after incubation for 2 days with exosome from RANKL-induced RAW 264.7 cells were analyzed by qRT-PCR. (**n**) Representative images of Alp staining of osteoblasts after incubation with exosome from RANKL-induced RAW 264.7 cells for 6 days, The culture medium was replaced for fresh mediun every 2 days. The osteoblasts were transfected with *Epha2* siRNA or its negative control. (**o**) RhoA activities in MC3T3-E1 cells after incubation with exosomes (5 μg/ml) secreted from osteoclasts were analysed by western blot. The data represent the mean ± SEM of three independent experiments. **P*<0.05, ***P*<0.01.
