# Supplementary material for: Osteoclast-derived microRNA-containing exosomes selectively inhibit osteoblast activity
Source: Cell Discov. 2016 May 31;2:16015–. doi: 10.1038/celldisc.2016.15 (PMC4886818; doi:10.1038/celldisc.2016.15)
Supplement: Supplementary Figure S5 [file celldisc201615-s5.pdf]

Supplementary Figure 5

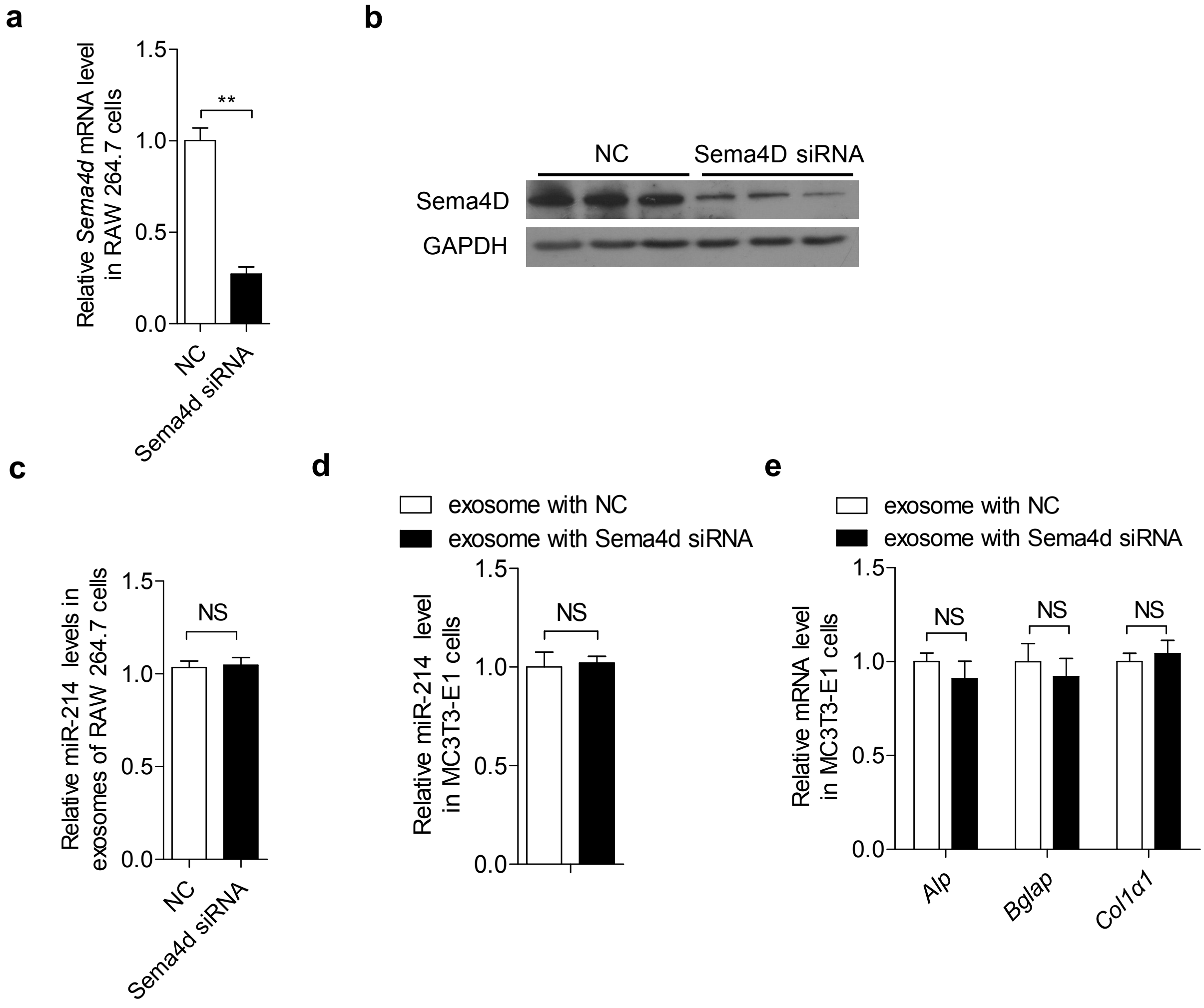

Supplementary Figure 5. Sema4D is not involved in the recognition of exosomes by osteoblasts.

(**a**) The efficiency of *Sema4d* siRNA. *Sema4d* mRNA level in RANKL-induced RAW 264.7 cells after transfection with *Sema4d* siRNA was analyzed by qRT-PCR. *Sema4d* mRNA levels were normalized to *Gapdh*. (**b**) Sema4D protein level after transfection with *Sema4d* siRNA was analyzed by western blot. (**c**) miR-214 levels in exosomes from *Sema4d* siRNA transfected RAW 264.7 cells. miR-214 levels were normalized to *RNU6*. (**d**) miR-214 levels in MC3T3-E1 cells after incubation for 2 days with exosomes from *Sema4d* siRNA transfected RAW 264.7 cells. miR-214 levels were normalized to *RNU6*. (**e**) *Alp, Bglap* and *Col1α1* mRNA levels in MC3T3-E1 cells were analyzed after incubation for 2 days with exosomes from *Sema4d* siRNA transfected RAW 264.7 cells by qRT-PCR. The mRNA levels were normalized to *Gapdh*. The data represent the mean ± SEM of three independent experiments. **P*<0.05, ***P*<0.01, NS, not significant.
