# Supplementary material for: Osteoclast-derived microRNA-containing exosomes selectively inhibit osteoblast activity
Source: Cell Discov. 2016 May 31;2:16015–. doi: 10.1038/celldisc.2016.15 (PMC4886818; doi:10.1038/celldisc.2016.15)
Supplement: Supplementary Figure S6 [file celldisc201615-s6.pdf]

Supplementary Figure 6

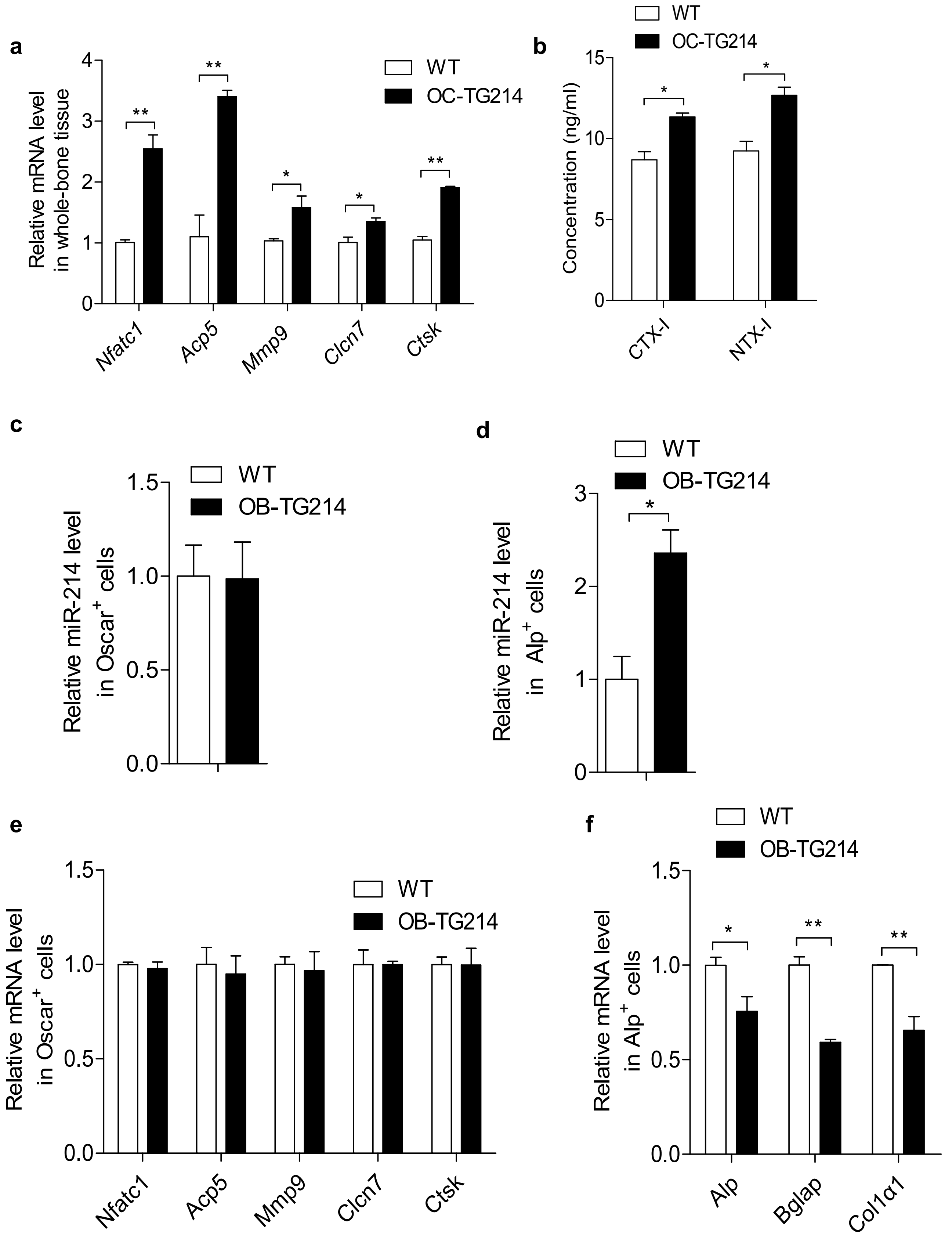

Supplementary Figure 6. Changes in osteoclast and osteoblast activities in OC-TG214 and OB-TG214 mice.

(**a**) *Nfatc1*, *Acp5*, *Mmp9*, *Clcn7* and *Ctsk* mRNA levels in whole-bone tissues of WT and OC-TG214 mice were analyzed by qRT-PCR. The PCR products were normalized to Gapdh. (**b**) Serum CTX-I and NTX-I levels in WT and OC-TG214 mice were analyzed by ELISA. n=3. (**c,d**) miR-214 levels in $Oscar^+$ cells and Alp^+ cells from OB-TG214 mice were analyzed by qRT-PCR. (**e,f**) *Nfatc1*, *Acp5*, *Mmp9*, *Clcn7* and *Ctsk* mRNA levels in $Oscar^+$ cells and *Alp*, *Bglap*, *Col1α* mRNA levels in Alp^+ cells from OB-TG214 mice were analyzed by qRT-PCR. The PCR products were normalized to *Gapdh*. The data represent the mean ± SEM of 3 experiments in triplicate; *$P<0.05$, **$P<0.01$.
