# Supplementary material for: Osteoclast-derived microRNA-containing exosomes selectively inhibit osteoblast activity
Source: Cell Discov. 2016 May 31;2:16015–. doi: 10.1038/celldisc.2016.15 (PMC4886818; doi:10.1038/celldisc.2016.15)
Supplement: Supplementary Figure S7 [file celldisc201615-s7.pdf]

Supplementary Figure 7

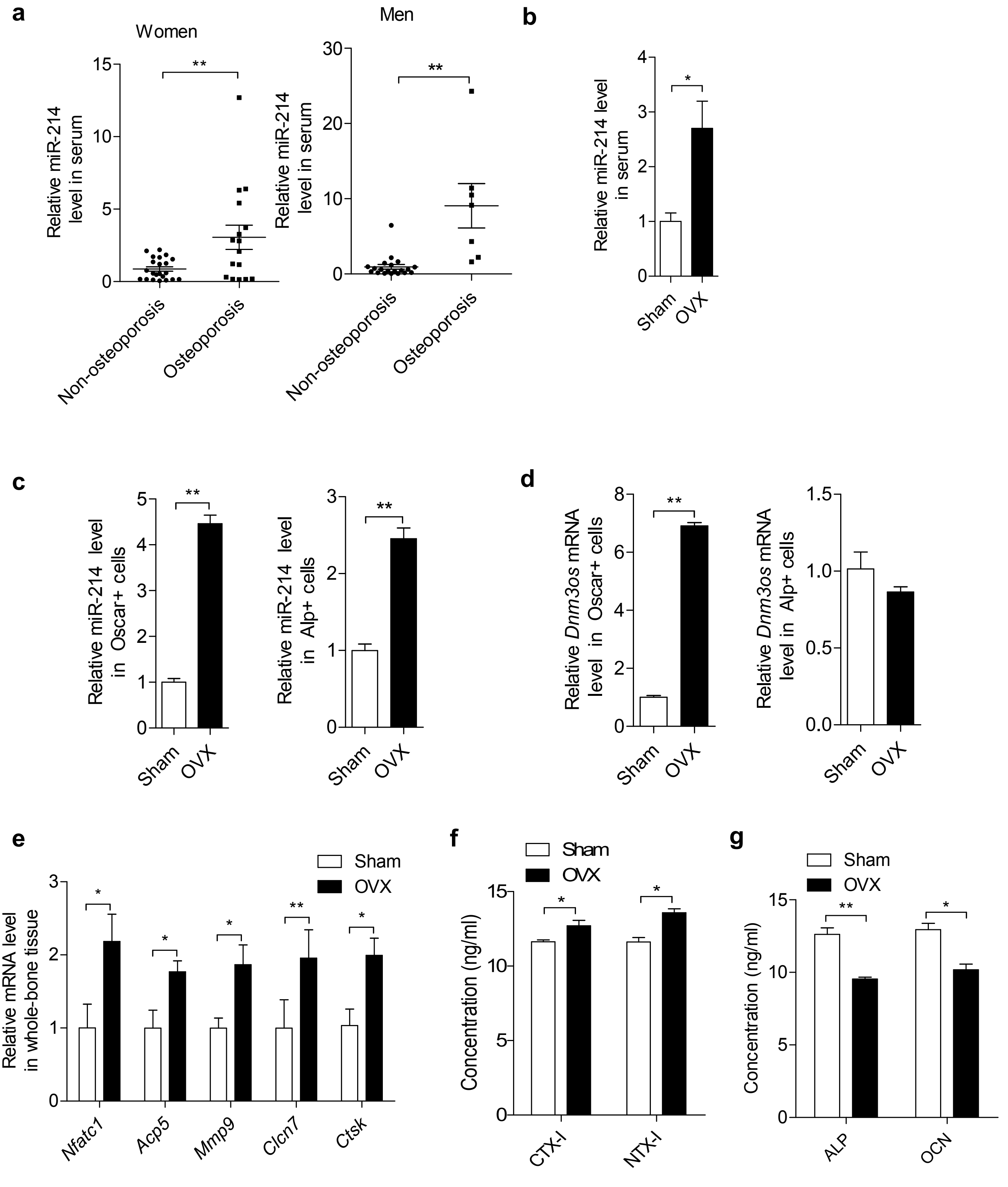

Supplementary Figure 7. Changes in miR-214 levels in osteoporotic patients, non-osteoporotic patients, OVX mice, and sham mice.

(**a**) QRT-PCR analysis of circulating miR-214 in serum (normalized to cel-miR-39) from osteoporosis vs non-osteoporosis patients. Non-osteoporosis women, n=22, non-osteoporosis men, n=16, osteoporosis women, n=20, osteoporosis men, n=7. (**b**) QRT-PCR analysis of miR-214 levels in serum (normalized to cel-miR-39) of sham and OVX mice. Sham, n=6, OVX, n=6. (**c**) miR-214 levels in Oscar$^+$ cells and Alp$^+$ cells from sham and OVX mice. (**d**) The change of *Dnm3os* mRNA level in Oscar$^+$ cells and Alp$^+$ cells from sham and OVX mice. *Dnm3os* mRNA level was analyzed by qRT-PCR and normalized to *Gapdh*. (**e**) QRT-PCR analysis the expression of *Nfatc1, Acp5, Mmp9, Clcn7 and Ctsk* in whole-bone tissues of OVX and sham mice. Sham, n=6, OVX, n=6. (**f, g**) Serum CTX-I, NTX-I, ALP, and OCN levels in OVX and sham mice. n=3. The PCR products were normalized to *Gapdh*. The data represent the mean ± SEM. *P<0.05, **P<0.01.
