# Supplementary material for: Osteoclast-derived microRNA-containing exosomes selectively inhibit osteoblast activity
Source: Cell Discov. 2016 May 31;2:16015–. doi: 10.1038/celldisc.2016.15 (PMC4886818; doi:10.1038/celldisc.2016.15)
Supplement: Supplementary Figure S8 [file celldisc201615-s8.pdf]

Supplementary Figure 8

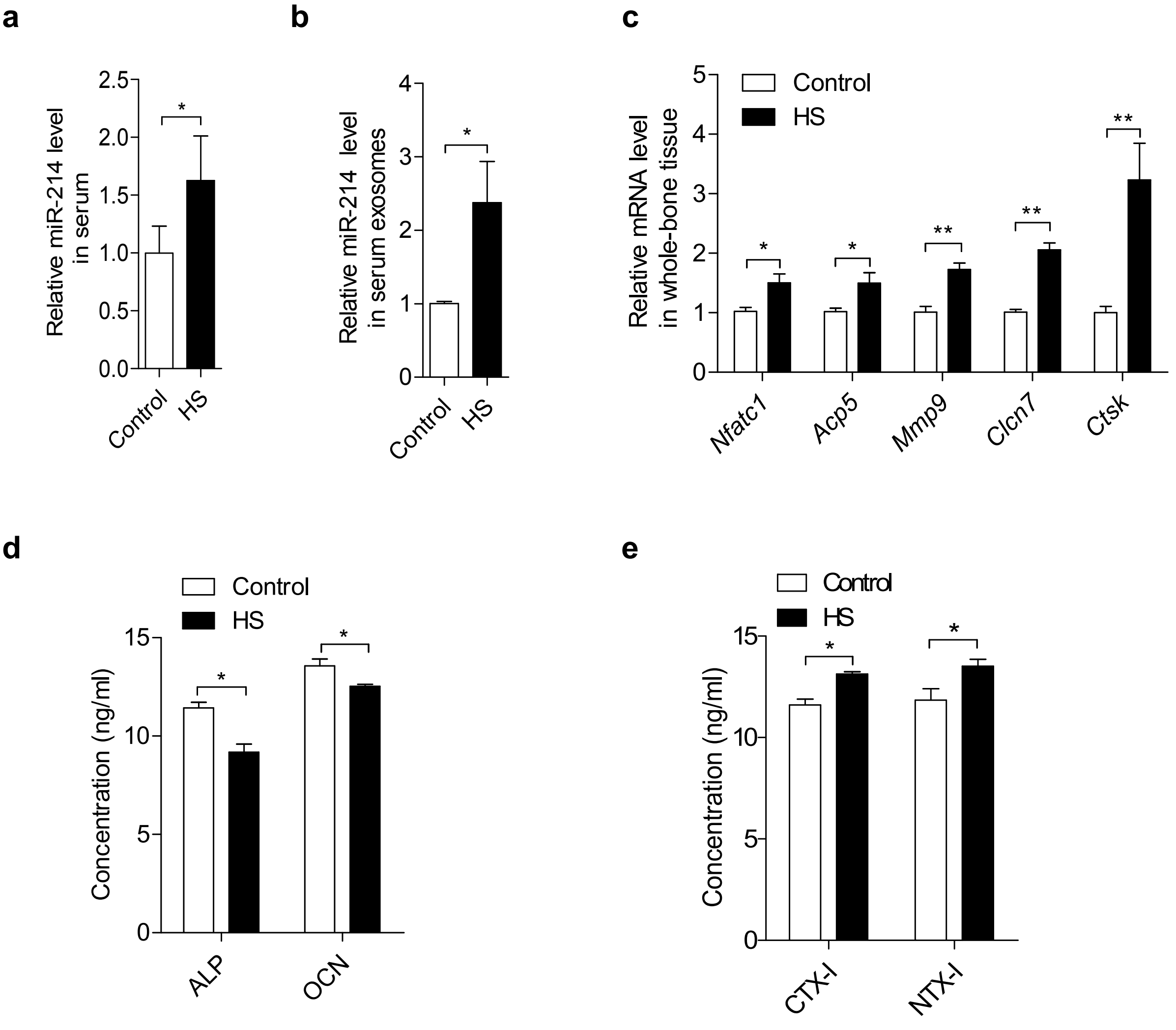

Supplementary Figure 8. Changes in circulating miR-214 levels in exosomes from hind-limb suspended mice and their inhibitory role on osteoblast activity.

(**a**) qRT-PCR analysis of miR-214 levels in serum of HS and control mice. Control, n=3, HS, n=5. The PCR products were normalized to cel-miR-39. (**b**) qRT-PCR analysis of miR-214 levels in exosomes from serum of HS and control mice. The PCR products were normalized to *RNU6*. (**c**) *Nfatc1*, *Acp5*, *Mmp9*, *Clcn7* and *Ctsk* mRNA levels in whole-bone tissues of HS and Control mice. (**d,e**) Serum ALP, OCN, CTX-I and NTX-I levels in HS and control mice were analyzed by ELISA. n=3. The data represent the mean ± SEM. **P*<0.05, ***P*<0.01.
