# Supplementary material for: Osteoclast-derived microRNA-containing exosomes selectively inhibit osteoblast activity
Source: Cell Discov. 2016 May 31;2:16015–. doi: 10.1038/celldisc.2016.15 (PMC4886818; doi:10.1038/celldisc.2016.15)
Supplement: Supplementary Figure S9 [file celldisc201615-s9.pdf]

Supplementary Figure 9

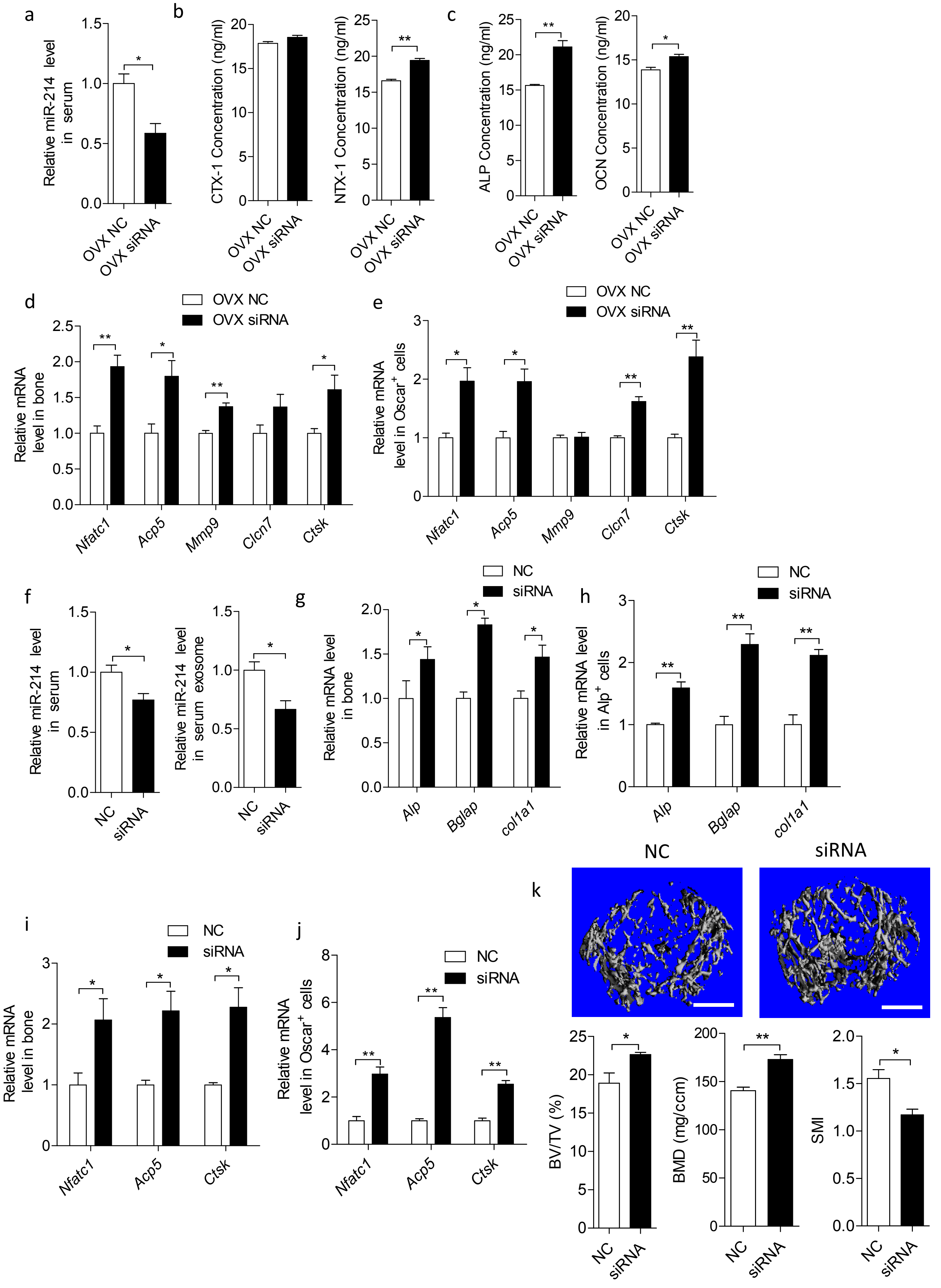

Supplementary Figure 9 Inhibition of exosome release by down-regulation of Rab27a attenuates the inhibitory role of miR-214 on osteoblast activity *in vivo*.

(**a**) qRT-PCR analysis of miR-214 level in serum (normalized to cel-miR-39) from OVX NC and OVX siRNA mice. MiR-214 levels were normalized to *RNU6*. (**b,c**) ELISA analysis of serum CTX-1,NTX-1,ALP, OCN levels in OVX NC and OVX siRNA mice. (**d,e**) *Nfatc1*, *Acp5*, *Mmp9*, *Clcn7* and *Ctsk* mRNA levels in whole-bone tissues and Oscar$^+$ cells of OVX NC and OVX siRNA mice were analyzed by qRT-PCR. OVX NC , n=4, OVX siRNA , n=4. (**f**) qRT-PCR analysis of miR-214 level in serum (normalized to cel-miR-39) and serum exosomes (normalized to *RNU6*) from NC and siRNA treated control mice. NC, control mice injected with *Rab27a* siRNA negative control; siRNA, control mice injected with *Rab27a* siRNA. (**g**) qRT-PCR analysis of *Alp*, *Bglap* and *Col1α1* mRNA in whole bone tissues of NC and siRNA mice. (**h**) qRT-PCR analysis of *Alp*, *Bglap* and *Col1α1* mRNA in Alp$^+$ cells of NC and siRNA mice. (**i**) qRT-PCR analysis of *Nfatc1*, *Acp5* and *Ctsk* mRNA in whole bone tissues from NC and siRNA treated mice. (**j**) qRT-PCR analysis of *Nfatc1*, *Acp5* and *Ctsk* mRNA levels in Oscar$^+$ cells from NC and siRNA treated mice. (**k**) Representative images showing three-dimensional trabecular architecture by microCT reconstruction in the distal femurs. BV/TV, the ratio of bone volume to tissue volume. BMD, Bone Mineral Density, SMI, Structure Model Index. Scale bars, 1 mm. The data represent the mean ± SEM of three independent experiments. *P<0.05, **P<0.01.
