# Supplementary material for: Osteoclast-derived microRNA-containing exosomes selectively inhibit osteoblast activity
Source: Cell Discov. 2016 May 31;2:16015–. doi: 10.1038/celldisc.2016.15 (PMC4886818; doi:10.1038/celldisc.2016.15)
Supplement: Supplementary Table S1 [file celldisc201615-s10.pdf]

Supplementary Table 1. miRNA analysis in exosomes released from RAW 264.7 cells with or without RANKL induction. Results are based on the miRNA profiling of three different samples from each type of exosome.

**Exosome from RAW 264.7 cells (12 miRNAs)**
miR-10a-5p, miR-15b-5p, miR-25-5p, miR-30e-3p, miR-103-3p, miR-139-3p, miR-192-3p, miR-365-2-5p, miR-374c-3p, miR-532-5p, miR-1198-5p, miR-3107-5p
**Both in two kinds of exosomes (overlapped expression) (70 miRNAs)**
let-7a-5p, let-7b-5p, let-7c-5p, let-7d-5p, let-7d-3p, let-7e-5p, let-7f-5p, let-7g-5p, let-7i-5p, miR-7a-5p, miR-10b-5p, miR-16-5p, miR-21a-5p, miR-22-3p, miR-23a-3p, miR-23b-3p, miR-26a-5p, miR-26b-5p, miR-27a-3p, miR-27b-3p, miR-30a-5p, miR-30b-3p, miR-30c-5p, miR-30d-5p, miR-30e-5p, miR-92a-3p, miR-92a-1-5p, miR-98-5p, miR-99a-5p, miR-99b-5p, miR-125a-5p, miR-127-3p, miR-140-3p, miR-142-5p, miR-143-3p, miR-146a-5p, miR-146b-5p, miR-148a-3p, miR-151-5p, miR-151-3p, miR-155-5p, miR-181a-5p, miR-181b-5p, miR-182-5p, miR-183-5p, miR-186-5p, miR-191-5p, miR-192-5p, miR-199a-3p, miR-214-3p, miR-221-3p, miR-222-3p, miR-320-3p, miR-378a-3p, miR-423-5p, miR-423-3p, miR-484, miR-671-3p, miR-702-5p, miR-1943-5p, miR-3107-3p, miR-3535, miR-5099, miR-6236
**Exosome from RANKL-induced RAW 264.7 cells (3 miRNAs)**
miR-101c, miR-125b-5p, miR-130b-3p
