# Supplementary material for: Osteoclast-derived microRNA-containing exosomes selectively inhibit osteoblast activity
Source: Cell Discov. 2016 May 31;2:16015–. doi: 10.1038/celldisc.2016.15 (PMC4886818; doi:10.1038/celldisc.2016.15)
Supplement: Supplementary Table S2 [file celldisc201615-s11.pdf]

Supplementary Table 2. Clinical features of osteoporotic and non-osteoporotic subjects recruited for analysis of circulating miR-214 levels.

Non-osteoporotic women	Age	T score for BMD at Spine	Osteoporotic women	Age	T score for BMD at Spine
1	57	-2.2	1	76	-2.7
2	68	-1.3	2	62	-3.2
3	71	-2.1	3	56	-3.8
4	66	-1.8	4	70	-3.0
5	84	-2.3	5	69	-2.5
6	62	0.1	6	90	-2.5
7	82	1.7	7	64	-2.5
8	66	-1.0	8	79	-2.6
9	55	-0.9	9	58	-3.5
10	57	-1.9	10	57	-3.8
11	78	-2.0	11	71	-3.6
12	62	0.2	12	71	-3.6
13	55	0.1	13	78	-3.5
14	71	-0.4	14	60	-3.5
15	63	-0.5	15	72	-2.9
16	82	-0.5	16	64	-3.5
17	48	-0.5			

18	78	-1.0			
19	66	-1.8			
20	72	-1.4			
21	82	-1.8			
22	84	-2.0			
**Non-osteoporotic men**	**Age**	**T score for BMD at Hip Joint**	**Osteoporotic men**	**Age**	**T score for BMD at Hip Joint**
1	74	-1.8	1	62	-2.9
2	71	-1.8	2	68	-3.2
3	56	-1.3	3	61	-3.4
4	74	-1.8	4	68	-3.1
5	57	-0.3	5	92	-3.3
6	57	-1.1	6	50	-3.1
7	69	-1.2	7	80	-3.4
8	74	-1.1			
9	51	-1.6			
10	66	-1.4			
11	64	-1.1			
12	51	0.7			
13	61	0.6			

14	79	0.5			
15	74	-0.3			
16	56	-0.5			
17	73	-0.9			
18	56	-1.1			
19	63	-1.6			
20	61	-2.4			
